# Profiling of Transcriptome-Wide N6-Methyladenosine (m6A) Modifications and Identifying m6A Associated Regulation in Sperm Tail Formation in *Anopheles sinensis*

**DOI:** 10.3390/ijms23094630

**Published:** 2022-04-22

**Authors:** Congshan Liu, Jianping Cao, Haobing Zhang, Jiatong Wu, Jianhai Yin

**Affiliations:** National Institute of Parasitic Diseases, Chinese Center for Disease Control and Prevention (Chinese Center for Tropical Diseases Research), NHC Key Laboratory of Parasite and Vector Biology, WHO Collaborating Center for Tropical Diseases, National Center for International Research on Tropical Diseases, Shanghai 200025, China; liucs@nipd.chinacdc.cn (C.L.); caojp@chinacdc.cn (J.C.); zhanghaobing2@163.com (H.Z.); wujt@nipd.chinacdc.cn (J.W.)

**Keywords:** *Anopheles sinensis*, epigenetics, m6A, sex-specific, spermatogenesis, sperm tail

## Abstract

Recent discoveries of reversible N6-methyladenosine (m6A) methylation on messenger RNA (mRNA) and mapping of m6A methylomes in many species have revealed potential regulatory functions of this RNA modification by m6A players—writers, readers, and erasers. Here, we first profile transcriptome-wide m6A in female and male *Anopheles sinensis* and reveal that m6A is also a highly conserved modification of mRNA in mosquitoes. Distinct from mammals and yeast but similar to *Arabidopsis thaliana*, m6A in *An. sinensis* is enriched not only around the stop codon and within 3′-untranslated regions but also around the start codon and 5′-UTR. Gene ontology analysis indicates the unique distribution pattern of m6A in *An. sinensis* is associated with mosquito sex-specific pathways such as tRNA wobble uridine modification and phospholipid-binding in females, and peptidoglycan catabolic process, exosome and signal recognition particle, endoplasmic reticulum targeting, and RNA helicase activity in males. The positive correlation between m6A deposition and mRNA abundance indicates that m6A can play a role in regulating gene expression in mosquitoes. Furthermore, many spermatogenesis-associated genes, especially those related to mature sperm flagellum formation, are positively modulated by m6A methylation. A transcriptional regulatory network of m6A in *An. sinensis* is first profiled in the present study, especially in spermatogenesis, which may provide a new clue for the control of this disease-transmitting vector.

## 1. Introduction

China was certified as a malaria-free country on 30 June 2021 (https://www.who.int/news/item/30-06-2021-from-30-million-cases-to-zero-china-is-certified-malaria-free-by-who, accessed on 30 June 2021), but the risk of re-establishment of malaria transmission in China cannot be neglected, mainly due to the fact that nearly 3000 malaria cases imported yearly [1] and the widespread distribution of malaria mosquito vectors, especially the most widely distributed species of *Anopheles sinensis* [2,3], which is considered to be a competent vector for *Plasmodium vivax* [4], and a potential vector for *P. falciparum* according to the positive susceptibility experiments in the laboratories [2,4]. Moreover, introduced vivax malaria cases have been reported in Liaoning province, Hunan province, and Yunnan border areas, respectively, which were speculated to be the secondary transmission caused by imported vivax malaria cases from Southeast Asia [5,6,7]. In addition, *An. sinensis* is also the vector that transmits pathogens severely impacting global human health, such as lymphatic filariasis [8,9], *Japanese encephalitis virus* [10], and *Rickettsia felis* [11]. However, available tools are not sufficient for mosquito control, which is the mainstay approach for diseases prevention and control. Thus, new strategies instead of traditional measures to control vectors are urgently needed [12].

Targeting mosquito physiological developments and physical behaviors in the life cycle are the key aspects to hindering disease transmission. Recently, epigenetic inheritance has been considered to be fundamentally important in cellular differentiation, neuronal functions, sex determination, and developmental plasticity of insects [13,14,15,16,17,18,19,20,21,22]. As one of epigenetics, N6-methyladenosine (m6A) is the most prominent modification of eukaryotic messenger RNA (mRNA) [23,24], including mammals, plants, *Drosophila*, and yeast [14,25,26,27]. By regulating mRNA fate, the cooperation of writers, readers, and erasers plays diverse roles in organism and diseases development [24,28]. Usually, m6A is installed by methyltransferases METTL3/METTL14 as the catalytic core, with some important co-factors including Wilms-tumor-1-associated protein (WTAP), Vir-like m6A methyltransferase associated (VIRMA), RNA binding motif 15 (RBM15), Zinc-finger CCH domain-containing protein (ZC3H13), and HAKAI [24]. Moreover, m6A was shown to control several physiological processes by regulating pre-mRNA splicing, mRNA decay, and translation, not only via conserved YTH protein family members mostly but also by changing the RNA secondary structures or “RNA switches” to alter the binding of some RNA interacting proteins and their recognition sites [29,30]. In addition, m6A is dynamically regulated in mammals by oxidative demethylation via the activity of two demethylases, fat mass and obesity-associated factors (FTOs) and AlkB homologue 5 (ALKBH5) [31,32].

The function and mechanism of m6A in modifying *Drosophila* mRNA have been studied in insects [14,15,16,17,18]. For instance, *Drosophila* retained the conserved writer complex (Ime4, Mettl14, Vir, Nito, Fl(2)d) and YTH-domain family proteins (YT521-B, CG6422), but no corresponding demethylases have been identified so far [30]. Flightless and neuronal function defects of *Drosophila* resulted from the knock-out of Ime4 were verified that brain functions were regulated by neuronal mRNA with m6A modification [16,17,23]. Furthermore, Ime4 and Mettl14 can also regulate the sex determination of *Drosophila* by controlling the splicing of the Sxl [15,16,18]. Recently, *Drosophila* Ime4 was found to be potentially involved in the establishment and maintenance of the somatic cyst-cell permeability barrier in spermatogenesis [20]. In other insects, m6A was found to regulate insecticide resistance in *Bemisia tobaci* [21] and diapause-related genes in silkworms [22] and impact caste differentiation and larval development in honey bees [20,33]. However, the biological roles of m6A mRNA in other insects, including mosquitoes, remain largely unknown.

To further investigate the functions of m6A on mRNA and to facilitate future studies of m6A in mosquitoes, we first report here transcriptome-wide m6A profiling in female and male *An. sinensis*. This study confirmed that m6A is a highly conserved RNA modification on mRNA across female and male *An. sinensis*. Intriguingly, m6A in *An. sinensis* is enriched not only around the stop codon and within 3′UTRs, such as that in yeast and mammalian systems, but also around the start codon, as that in *Drosophila* and *Arabidopsis thaliana*. Furthermore, a positive correlation between m6A deposition and mRNA levels indicates a regulatory role of m6A in the gene expression of female and male mosquitoes. More importantly, m6A is found to play potential roles in spermatogenesis, especially in the sperm tail formation of *An. sinensis*.

## 2. Results

### 2.1. Conserved mRNA m6A Toolkit in Anopheles sinensis and Other Anopheles Species

**Figure 1:**
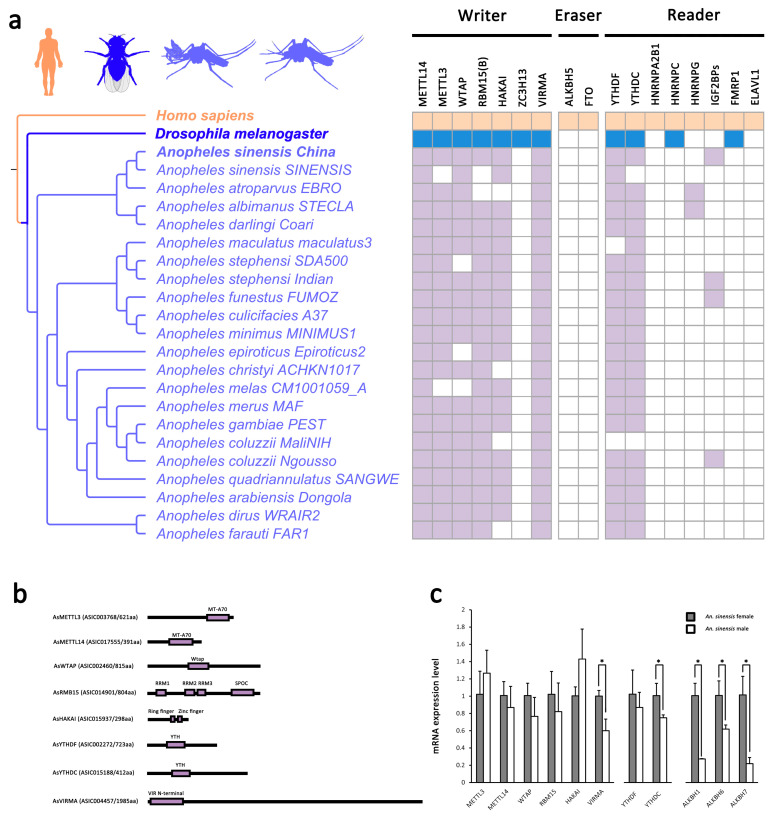
The toolkit of mRNA m6A modification in *An. sinensis* and *Anopheles* spp. (**a**) The distribution of mRNA m6A components in *Anopheles* spp. (**b**) Domain-structure of m6A writer and readers in *An. sinensis*. (**c**) The gene expression level of mRNA m6A components in female and male *An. sinensis* (*: *p* < 0.05).

Putative orthologs for the writer complex and YTH domain-containing proteins (Figure 1a, Supplementary File S1) were found in *An. sinensis* through a sequence-similarity approach using reciprocal best BLAST. Moreover, the conserved domain and phylogenetic analyses (Supplementary File S1) ascertained these orthology relationships. *Anopheles* spp. retains a core methyl transferase complex (METTL3, METTL14, and WTAP) and other components, such as HAKAI, RBM15 and VIRMA, and YTHDC and YTHDF as potential m6A readers (Figure 1b). However, no corresponding eraser enzymes (FTO and ALKBH5) were identified in *Anopheles* spp. genomes (Figure 1a,b, Supplementary File S1).

There was no significant difference in gene expressions of core methyltransferase complex between female and male *An. sinensis*, while that of VIRMA and YTHDCs were significantly lower in the males compared to the females (Figure 1c). Although no ALKBH5 and FTO were identified in *Anopheles* spp., the gene expression levels of other ALKBHs demethylases, which can potentially compensate for the absence of ALKBH5 and FTO, were significantly lower in the males than the females (Figure 1c).

### 2.2. Mapping of m6A Modification on mRNA in Male and Female Anopheles sinensis

#### 2.2.1. m6A Is Abundant and Conserved in Both Female and Male *An. sinensis*

**Figure 2:**
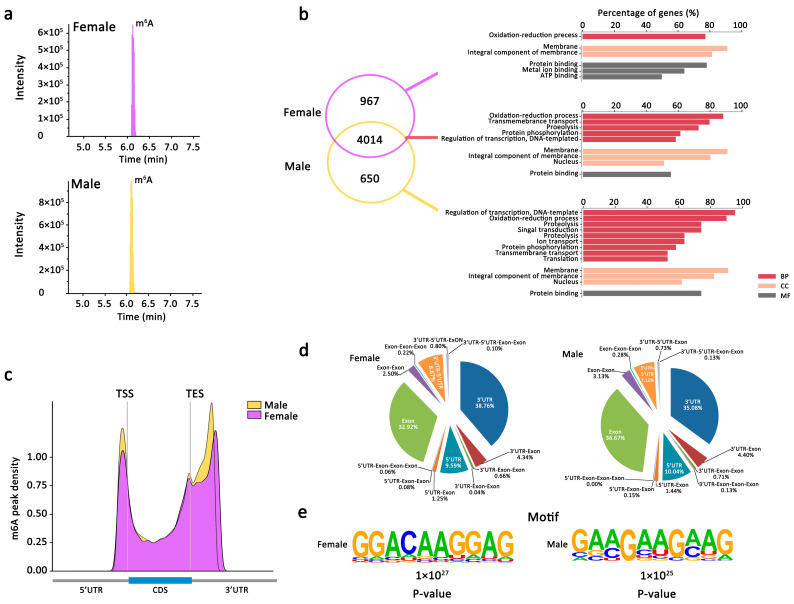
Overview of m6A methylome in female and male *An. sinensis*. (**a**) mRNA m6A in female and male *An. sinensis* detected by mass spectrometry. (**b**) Numbers of sex-specific and common m6A peaks genes and GO enrichment. (**c**) Accumulation of m6A-IP reads along transcripts. Each transcript was divided into three parts: 5′UTRs, CDS and 3′UTRs. (**d**) The distribution of m6A peaks within different gene contexts. (**e**) Sequence motifs with the most m6A peaks.

The existence of m6A modification on mRNA of female and male *An. sinensis* was preliminarily identified by mass spectrometry (Figure 2a). To further obtain the transcriptome-wide m6A map, both female and male *An. sinensis* were interrogated using m6A-targeted antibody coupled with high-throughput sequencing. In total, 6756 m6A peaks (fold change ≥ 2.0) representing the transcripts of 4981 genes in females, and 6289 m6A peaks representing the transcripts of 4664 genes in males, were identified, respectively (Figure 2b). Among them, 4014 m6A tagged genes were detected within both female and male *An. sinensis*, most of which were related to the oxidation-reduction process, membrane, and protein binding. Moreover, there were 967 female-specific m6A peak genes related to metal ion binding and ATP binding and 650 male-specific m6A peak genes involved in the regulation of transcription and DNA template (Figure 2b). Additionally, in *An. sinensis*, there were abundant genes with sex-specific m6A peak without known functions, indicating their species- and sex-specific functions associated with m6A modification. Above all, it was estimated that the *An. sinensis* transcriptome contained 0.7–0.8 m6A peaks per expressed transcript (active expressed transcript FPKM ≥ 2) (Table S1).

#### 2.2.2. m6A Peaks Positions in Female and Male *An. sinensis*

After analyzing the distribution of m6A in the whole transcriptome for both female and male m6A-IP and non-IP (input) samples, it was found the m6A-IP reads were highly enriched around the 5′UTR, start codon, stop codon, and within 3′UTR (Figure 2c). It was also found the peak callings of female samples were distributed almost equally in transcription start (TSS) and end (TES) sites, while more peak callings were detected in the TES regions than in TSS regions in male *An. sinensis* (Figure 2c). Moreover, the global hyper-methylation of m6A was induced in male *An. sinensis* compare to females. The subgroups of genes were classified according to their detailed m6A peaks positions (Figure 2d). Most of the m6A sites were found at 3′-UTR (35.08–38.76%), exon (32.92–36.67%), and 5′-UTR (7.12–8.67%). In addition, there were m6A peaks at both 5′UTR and 3′UTR in the females (8.67%) and the males (7.12%).

#### 2.2.3. m6A Peaks Motifs in Female and Male *An. sinensis*

Top 1000 significant peaks were used to determine whether the m6A peaks in female and male *An. sinensis* contained the m6A consensus sequences. A core motif sequence of GGACAAGGAGG was identified in the females, showing the conserved pattern of the RRACH motif (Figure 2e). Meanwhile, a specific GAA/CGAAGA/CAG motif was observed in the male m6A peak genes (Figure 2e).

#### 2.2.4. Gene Expression and Important Biological Pathways of m6A-Containing mRNAs Genes

**Figure 3:**
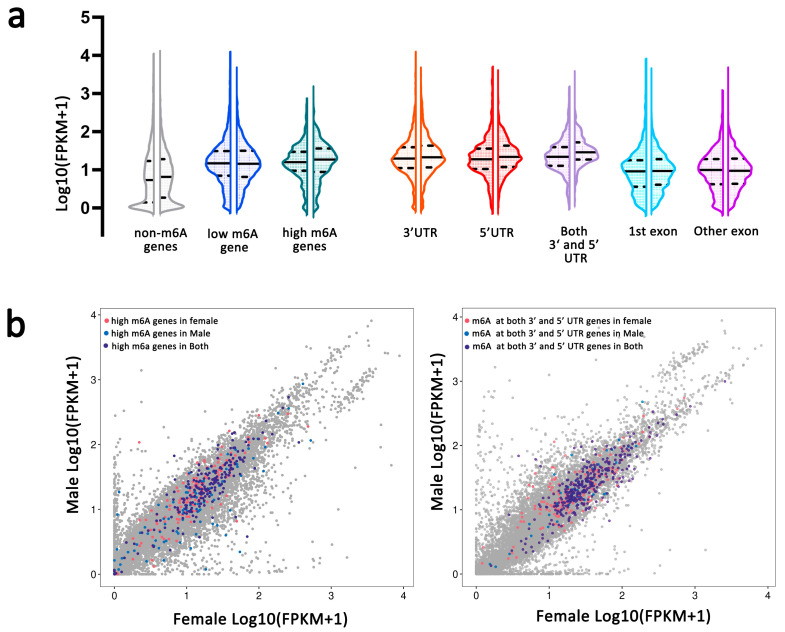

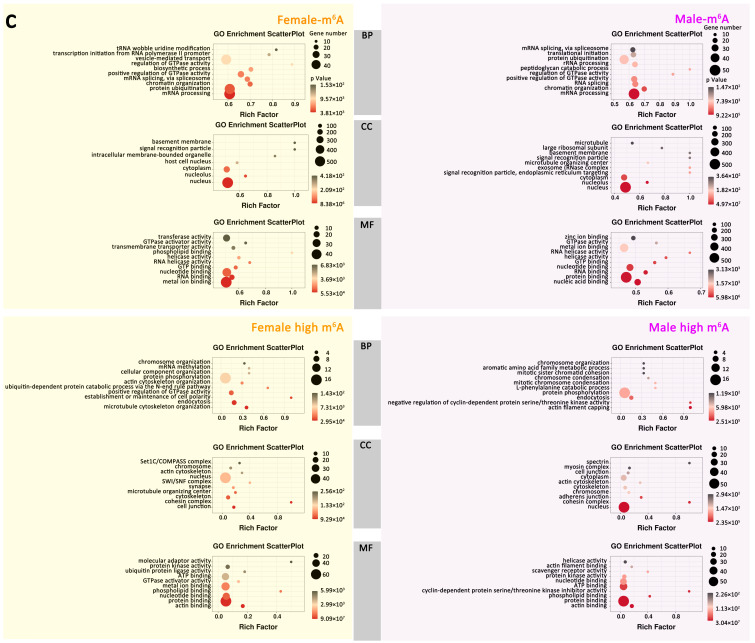
m6A-containing mRNAs related to gene expression and important biological pathways in female and male *An. sinensis*. (**a**) The mRNA expression levels in females (left) and males (right) containing m6A peaks. Genes were divided into three categories according to the numbers of m6A sites in each gene. non-m6A gene (number of m6A site = 0), low m6A gene (number of m6A site < 3), high m6A gene (number of m6A site ≥ 3). Genes were also divided into five categories (3′ UTR, 5′ UTR, both 3′ and 5′ UTR, 1st exon and other exon) according to the annotation of the m6A peak in each gene. (**b**) Differentially expressed genes in female and male *An. sinensis*. Genes with high m6A modification and genes with m6A peaks at both 3′ and 5′ UTR are highlighted with colorful dots. (**c**) GO enrichment of sex-specific m6A genes in female and male *An. sinensis*. Female-m6A, m6A genes in female; Male-m6A, m6A genes in male; female high m6A genes, genes with m6A sites ≥ 3 in females; Male high m6A genes, genes with m6A sites ≥ 3 in males.

The expression levels of genes with m6A modification were significantly higher than that of genes without m6A modification in both female and male *An. sinensis* (Kruskal–Wallis test, *p* < 0.001) (Figure 3a,b). Moreover, the expression of m6A modified genes at UTR regions had higher levels than those at exon regions or without m6A modification, respectively (Kruskal–Wallis test, *p* < 0.001) (Figure 3a,b).

Then, the enriched GO (gene ontology) was further predicted using the genes containing m6A in female and male mosquitoes to uncover potentially functional insights about m6A in *An. sinensis* (Figure 3c). It was found that these genes were enriched in tRNA wobble uridine modification, regulation of GTPase activity, basement membrane, signal recognition particle, and phospholipid-binding in the females, while they were enriched in peptidoglycan catabolic process, regulation of GTPase activity, basement membrane, signal recognition particle, exosome and signal recognition particle, endoplasmic reticulum targeting and RNA helicase activity in the males (Figure 3c). Importantly, in female *An. sinensis*, the high m6A-containing genes were associated with the establishment or maintenance of cell polarity and cohesion complex. In male *An. sinensis*, the high m6A-containing genes were involved in negative regulating of cyclin-dependent protein serine/threonine kinase activity, actin filament capping, spectrin, cohesion complex, and cyclin-dependent protein serine/threonine kinase inhibitor activity (Figure 3c).

#### 2.2.5. mRNA m6A Peaks with Different Abundance between Male and Female *An. sinensis*

**Figure 4:**
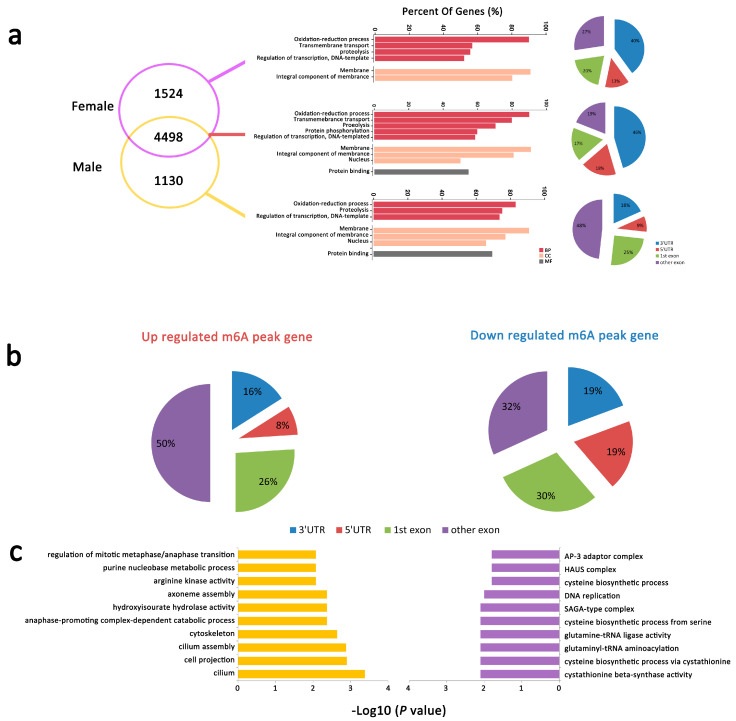
Differential m6A peaks in female and male *An. sinensis*. (**a**) The distribution of genes with differentially m6A peaks at different positions between female and male *An. sinensis* (male vs. female). (**b**) The distribution of up and down regulated m6A peak genes. (**c**) GO-enrichment analysis of genes with up-(**left**) and down-(**right**) regulated m6A peaks.

A different intensity was also found in m6A peaks in female and male *An. sinensis* (Figure 4a). Among them, 88 down-regulated m6A peaks in 86 genes and 55 up-regulated m6A peaks in 45 genes were found in the males (*p* < 0.05. FC ≥ or ≤ 2). The peaks in these genes were mostly identified in the exon, especially in other exons rather than the 1st exon (Figure 4b). GO analysis indicated that the genes with dynamic m6A peaks were enriched in several categories of fundamental biological functions; for example, cilium, cell projection, and cilium assembly were regulated by genes with up-regulated m6A peaks, and cysteine biosynthesis, cystathionine beta-synthase activity were regulated by genes with down-regulated m6A peaks (Figure 4c).

### 2.3. Different Gene Expression in Male and Female An. sinensis

**Figure 5:**
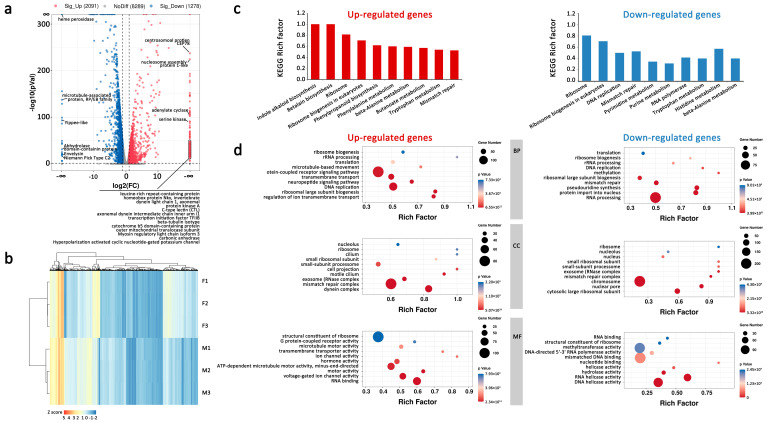
Differentially expressed genes in female and male *An. sinensis* (male vs. female). (**a**) Up-and down-regulated genes [log2(fold change) ≥ 1 and *p* < 0.05] between female and male *An. sinensis*. (**b**) Correlation of gene expression among three biological replicates. (**c**) Top ten KEGG enrichment of differentially expressed genes (*p* < 0.05). (**d**) GO enrichment of differentially expressed genes. BP, biological process; CC, cellular component; MF, molecular function.

A total of 3369 differentially expressed genes were identified between male and female *An. sinensis*, and 2091 genes were up-regulated, and 1278 were down-regulated, respectively (male vs. female) (Figure 5a,b). Among them, some sex-specific genes were identified, such as Heme peroxidase, Envelysin, Yippee-like, microtubule-associated protein, RP/EB family, Abhydrolase domain-containing protein, and Niemann-Pick Type C-2 were only actively expressed in female mosquitoes, while adenylate cyclase, leucine-rich repeat-containing protein, serine kinase, homeobox protein Nkx, invertebrate, dynein light chain 1, were specifically identified in male mosquitoes (Figure 5a). Up-regulated differentially expressed genes were dominated in the pathways of Indole alkaloid biosynthesis, betalain biosynthesis, ribosome and ribosome biogenesis in eukaryotes, while down-regulated differentially expressed genes were in the ribosome, ribosome biogenesis, and histidine metabolism (Figure 5c). In addition, up-regulated expressed genes were highly enriched in biological processes of rRNA procession, microtubule-based movement, neuropeptide signaling pathway, ribosomal large subunit biogenesis, and regulation of ion transmembrane transport; in cellular components of the ribosome, cilium, cell projection, motile cilium; and in molecular functions of transmembrane transporter activity and ion channel activity, respectively (Figure 5d). The down-regulated expressed genes were highly enriched in biological processes of DNA replication, methylation, pseudouridine synthesis, and protein import into the nucleus; in cellular components of small ribosomal subunit, small-subunit processome, exosome (RNase complex), mismatch repair complex, and nuclear pore; and in molecular function of nucleotide-binding (Figure 5d).

### 2.4. m6A Modification and Gene Expression in Female and Male An. sinensis

#### 2.4.1. Correlation of m6A Modification and Gene Expression

**Figure 6:**
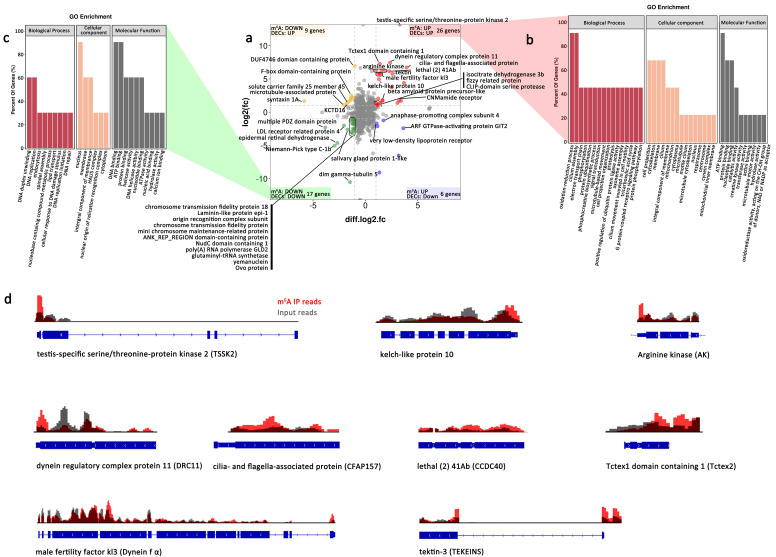
Correlation analysis of differentially modified m6A methylation and differentially expressed genes in female and male *An. sinensis* (male vs. female). (**a**) Relationship between m6A methylation and gene expression. (**b**) GO enrichment of down-regulated genes with m6A hypo-methylaiton. (**c**) GO enrichment of up-regulated genes with m6A hyper-methylaiton. (**d**) Reads abundance of m6A IP and input in male spermatogenesis-related genes in male *An. sinensis*.

After analyzing the transcriptome sequencing with or without an m6A marker, 54 differentially expressed genes with different m6A abundances in female and male *An. sinensis* (male vs. female) were identified (Figure 6a). Among them, 22 genes displayed m6A hyper-methylation and up-regulated expression (hyper-up), 9 genes had hyper-methylation and down-regulated expression (hyper-down), 6 genes displayed m6A hypo-methylation and up-regulated expression (hypo-up), and 17 genes had hypo-methylation and down-regulated expression (hypo-down) (Figure 6a, Table 1). Notably, hyper-up genes were mainly involved in the oxidation-reduction process, cilium assembly, cell projection, cytoskeleton, membrane and cilium, ATP/protein/nucleotide-binding (Figure 6b), while hypo-down genes were enriched in DNA repair-related process in the nucleus, with binding to DNA and ATP (Figure 6c).

#### 2.4.2. m6A Play Roles in Spermatogenesis Associated Genes in Male *An. sinensis*

The 54 differentially expressed genes with different m6A peaks were carefully annotated with corresponding information from model organisms (human, *D. melanogaster*, and *C. elegans*). Interestingly, it was noticed that 14 up-regulated genes involved in spermatogenesis, especially in the development of sperm flagellum, were potentially regulated by m6A modification on mRNA (Figure 6a,d).

##### m6A Associated with Spermatogenesis of Male *An. sinensis*

**Figure 7:**
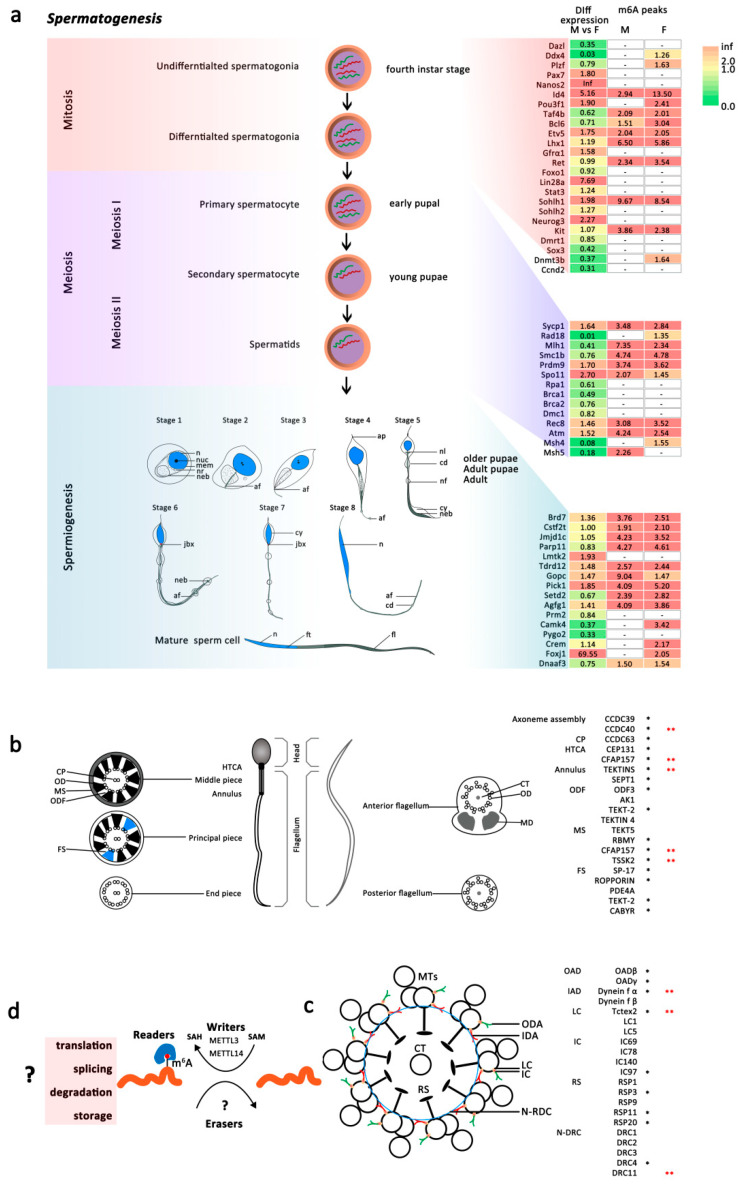
Spermatogenesis of male *An. sinensis* (male vs. female). (**a**) A schematic representation of different stages of spermatogenesis in mature mosquito sperm was produced. Diploid spermatogonia undergo an initial mitotic division to produce diploid primary spermatocyte, followed by meiosis I to produce haploid secondary spermatocytes. These secondary spermatocytes further divide through meiosis II and produce haploid spermatids. Mature sperm cells were developed from spermatids through the final stage called spermiogenesis. Eight developmental stages of mosquitoes were adapted from illustration of spermiogenesis in *Adeds aegypti* [34], n = nucleus, nuc = nucleolus, mem = membranes of the endoplasmic reticulum, nr = neutral red staining vacuole and granule, neb = mitochondrial nebenkern, af = axial filament, ap = cytoplasmic filament, jxb = juxtanuclear body, cd = cytoplasmic swellings or droplets, nl = lobes of the nebenkern filaments, nf = nebenkern filament, cy = cytoplasm, neb = nebenkern, ft = slightly forked or bifurcated posterior of the nucleus, fl = flagellum. The m6A-target genes in spermatogenesis were mapped to *An. sinensis* transcriptome and MeRIP-seq data. Heatmap (right) of the gene expression and m6A modification of regulatory components. FC, fold change. (**b**) The structure of the sperm tail and its associated proteins: mammalian (**left**) and *An. sinensis* (**right**) sperm. Schematic representation of the spermatozoa including the HTCA (head tail coupling apparatus), middle piece, annulus, principal, and end piece of the sperm tail in mammalian sperm. The cross section of the middle piece contains the axoneme (CP = central pair, OD = outer doublet microtubules), outer dense fibers (ODFs), mitochondrial sheath (MS). The cross section of the principal piece includes the axoneme, ODFs, and fibrous sheath (FS). The asonemal structure is retained in the end piece of sperm tail. Anterior and posterior flagellums are in mosquito sperm tail. The cross section of the anterior flagellum includes the axoneme and mitochondrial derivatives (MD). The axonemal structure was also retained in the posterior flagellum of sperm tail. CT = central tubule. Proteins associated with these structures are listed in the right side. Black *, m6A peaks in male or female *An. sinensis*, red **, different m6A peaks between male and female *An. sinensis*. (**c**) The structure and male specific proteins in axomeme of *An. sinensis* sperm flagellum. MTs = microtubules, ODA = outer dynein arm, IDA = inner dynein arm, LC = light chian, IC = intermediate chain, RS = radial spoke, N-RDC = Nexin–Dynein regulatory complex. Proteins associated with these structures are also listed in the right side. Black *, m6A peaks in male or female *An. sinensis*, red **, different m6A peaks between male and female *An. sinensis*. (**d**) Hypothesis of the regulatory roles of m6A mRNA modification during mosquito spermatogenesis.

Compared to the mammalian spermatogenesis with three stages, mosquitoes retained the conserved mitosis and meiosis process but had a unique spermiogenesis process undergoing eight stages to form the mature sperm (Figure 7a) [34]. Recently, m6A was reported to be involved in the entire process of spermatogenesis, and many m6A-target transcripts have been identified in male germ cells [35,36,37,38,39]. In the present study, the orthologs of genes coding these transcripts were also identified in *An. sinensis* genome. Importantly, many of these genes were expressed differentially between female and male mosquitoes, and there were m6A modifications on their mRNAs (Table S2, Figure 7a). However, no significant difference between male and female *An. sinensis* was found in the m6A peaks abundances in most genes.

##### m6A in Mature Sperm of Male *An. sinensis*

Unlike mammalians’, mosquitoes’ sperm is longer and more slender, with its head (nucleus) as wide as the tail (flagellum) [40,41]. The flagellum consists of two mitochondrial derivatives (MD), which extend the length of the flagellum, and an axoneme, a microtubular structure responsible for motility. Mosquito sperm axonemes have 19 microtubules arranged in two concentric circles of nine around a long central tubule (9  +  9  +  1) [40,41] (Figure 7b). Many mammalian sperm-tail-associated proteins in the main structures of sperm, such as axoneme, center pair (CP), outer dense fibers (ODFs), mitochondrial sheath (MS), HTCA (head tail coupling apparatus), and annulus, have been extensively documented, while no protein in axoneme and mitochondrial derivatives of mosquitoes have been systematically studied (Figure 7b).

Here, the orthologs of these functional proteins were identified in *An. sinensis* genome. Most of these genes were highly expressed in male mosquitoes and contained m6A modification on their mRNA. Particularly, significantly different m6A peak abundances in CCDC40, CFAP157, TEKTINS, TSSK2, Dynein fα, Tctex2, and DRC11 in the males were correlated with their high expression levels (Figure 7b,c, Table 1 and Table S3). Moreover, many male-specific or highly expressed genes identified using the sperm proteomics of *Aedes aegypti* [42] were also tagged with m6A modification in *An. sinensis* (Table S4). Among them, arginine kinase responsible for maintaining constant ATP level to support the movement of sperm flagellum, and mitochondrial isocitrate dehydrogenase subunit without known functions in mosquitoes, were potentially regulated by the higher m6A modified mRNA in the males, while CFAP57, DUF4746 domain-containing protein, and BTB/POZ domain-containing protein KCTD12 showed lower m6A modification levels (Figure 6a, Table S4). In addition, it is noticed that kelch-like protein 10 (Klhl10), as a substrate-specific adapter of a CUL3-based E3 ubiquitin-protein ligase complex [43], was also highly expressed in male mosquitoes and tagged with m6A modification (Figure 6a).

## 3. Discussion

Mosquito-borne diseases such as malaria, dengue, and Zika are spread by the bite of an infected female mosquito rather than the harmless nonbiting males; thus, control methods biasing the sex ratio of their offspring by reducing the number of blood-sucking females or converting them into harmless males, have long been sought [44,45,46,47]. Genetic elements such as sex-chromosome drives can distort sex ratios to produce unisex populations that eventually collapse, and advanced technological breakthroughs at driving maleness to achieve efficient sex-separation, the ultimate disease refractory phenotype [48,49,50], become possible and may represent efficient and self-limiting methods instead of the traditional vector control measures to effectively reduce and control the target mosquito populations, but the underlying molecular signals and mechanisms in illustrating the entire sex development pathway in mosquitoes remain unknown [50]. The identification of m6A mechanism components, the existence of m6A on mRNA by mass spectrometry, and the transcriptome-wide map of a mosquito species *An. sinensis* presented here provides a starting roadmap for uncovering m6A functions that may affect/control mosquito development in the future. The m6A players and m6A target genes, especially the ones that play important roles in sperm tail, might be the potential target for further mosquito and disease control.

The m6A mRNA modification originated from the last eukaryotic common ancestor and has been considered a conserved mechanism for regulating the gene expression and translation in eukaryotes [14,25,26,27]. However, the existence and biological roles of m6A on mRNA in most insects, including mosquitoes, remain largely unknown. Here, this modification was first revealed in mosquitoes. Firstly, the identification of putative orthologs of m6A writer complex and YTH domain-containing proteins in *Anopheles* spp. suggested that mRNA m6A machinery was conserved and important in *Anopheles* spp. Noticeably, *An. sinensis* retains most of the m6A players, including writer complex and readers. Consistent with *Drosophila* [30], no ALKBH5 and FTO were identified in *Anopheles* spp., but the presence of other ALKBHs demethylases might potentially compensate for the absence of ALKBH5 and FTO. Importantly, the existence of m6A RNA modification was further confirmed by LC-MS in both male and female *An. sinensis*. Moreover, it was estimated that the *An. sinensis* transcriptome contains 0.7–0.8 m6A peaks per expressed transcript, which is similar to that obtained in mammals and plants [25,26]. Above all, the m6A mechanism is highly conserved in mosquitoes and potentially plays a role in the biological functions of mosquitoes.

Also, the profiling of the transcriptome-wide m6A distributions in *An. sinensis* further uncovered the m6A modification characteristics, including m6A positions on mRNA, the relationships between m6A distribution and gene expressions, and regulation of the sex-specific functions. In contrast to mammals and yeast [25,27], m6A on *An. sinensis* mRNA was not only highly enriched around the stop codon and at 3′ UTR, but also around the start codon and at 5′ UTR, as that has been found in *Drosophila* [15] and *A. thaliana* [26], and they are bona fide m6A enrichment peaks rather than m6Am at the cap of mRNA [25,26,27]. It was surprising to find that the m6A pattern in *An. sinensis* was distinct from mammals and yeasts but resembled *A. thaliana*, which is evolutionarily distinct from *Amorphea* [51]. This evidence shows diverse m6A patterns in different insects [15,52], as well as those in plants [26,53]. It also indicated that although m6A is a conserved mechanism for regulating the gene expression and some aspects of biological processes, the development and realization of this mechanism are different in different species. Therefore, it is necessary to reveal the fine-tuning mechanism of m6A in distinct organisms in the future.

The present study showed that the expression levels of genes with m6A modification were significantly higher than genes without m6A modification in both female and male *An. sinensis*, indicating m6A is a way to positively regulate the gene expression. Particularly, similar to *Bombyx mori* [52] and *A. thaliana* [26], the m6A peaks at 5′ UTR and 3′ UTR were positively correlated with the overall up-regulation of mRNA expression levels in both female and male *An. sinensis*. However, this relationship contradicted the findings of a negative correlation between gene expression and m6A methylation around the stop codon and at 3′ UTRs in mammalian, yeast, and honey bee systems [19,25,27]. Hence, it is suggested that m6A reader proteins may play roles in recognizing m6A at 5′ UTRs or 3′ UTRs and subsequently affect the stability of the target mRNA and then directly impact translation through the methylation itself or through the reader proteins [26].

Furthermore, the correlation of the sex specific functions with the distribution and density of m6A methylation in *An. sinensis* transcriptome was also analyzed. Firstly, the m6A peak density was more abundant in male than in female *An. sinensis*. Given that no differences in gene expression levels of m6A core writer complex were found in female and male *An. sinensis* (Figure 1c), the distinct m6A abundances may be contributed by other differentially expressed m6A components, such as VIRMA and potential ALKBH demethylases, because their lower expression levels were positively correlated with the higher abundance of m6A in male mosquitoes. Secondly, different m6A motif patterns in female and male *An. sinensis* add the complexity of m6A modification in regulating sex-related functions. GO and KEGG analysis of the genes with m6A modification indicated its critical roles in regulating mosquito development, especially in the sex-related respect. For instance, the transcripts with abundant m6A were highly enriched in cilium-related genes in the males, while m6A in genes associated with the cysteine biosynthetic process were down-regulated. Intriguingly, after crosslink analysis of m6A peak abundances and gene expression levels, it was found that, out of 54 target genes, there were 14 genes associated with spermatogenesis, strongly indicating the relationship between m6A modification and up and down-regulated gene expressions involved in spermatogenesis.

Spermatogenesis is a highly sophisticated and complex process that can be divided into three stages: mitosis, meiosis, and spermiogenesis [35]. Mosquitoes retained the conserved mitosis and meiosis process but have a unique spermiogenesis process undergoing eight stages to form the mature sperm (Figure 7a) [34]. m6A modification has been reported to be involved in mammalian spermatogenesis [27,31,32,35,37,38,54,55,56,57,58,59,60]. For instance, m6A methyltransferase complexes have been verified to play important roles in mammalian spermatogonial stem cell differentiation, meiosis, and spermiogenesis [27,35,37,54,55]. Sperm motility in the mice was significantly reduced after double-knockout of METTL3 and METTL14, accompanied by flagella defects and abnormal sperm head [38]. Moreover, the highly expressed FTO in the mammalian testis and its relationship with male fertility indicates FTO plays an important role in spermatogenesis [31,32,56,57]. Additionally, YTH domain proteins can also affect spermatogenesis by controlling germline transition into meiosis, participating in the normal function of spermatogenic tubules, and regulating spermatogonia migration and proliferation [58,59]. Recently, many genes were identified to be under the regulation of m6A modification in spermatogonial differentiation, meiosis, spermiogenesis, and other processes [32,37,38,56,60]. These genes with m6A modification were also identified in the *An. sinensis* transcripts, suggesting their potential roles in mosquito spermatogenesis. However, the m6A peak abundances were not significantly different between male and female adult mosquitoes, as well as the expression levels. It may be attributed to the physiological process that spermatogenesis is mainly developed in mosquito larvae rather than in adult males (Figure 7a), the development stage of which we focused on in this study. However, in adult male *An. sinensis*, m6A modification was found to be abundant in sperm tail associated proteins, which are involved in sperm tail formation and specifically expressed in mature sperm. Even though the sperm structures between mammals and mosquitoes are different [40,41], the sperm-related genes in *An. sinensis* identified by bioinformatics methods showed the conservation and complexity of sperm genes in mosquitoes. Meanwhile, the identification of spermatogenesis-related m6A peaks in genes indicates that their transcript, splicing, translation, and storage are potentially regulated by m6A modification. Additionally, the high expression levels of some functional genes in mature sperm tails are correlated with their abundant m6A modification on mRNA.

## 4. Materials and Methods

### 4.1. Mosquito Rearing and Sample Preparation

*Anopheles sinensis* (China strain) were reared at 28 ± 2 °C, 70–75% humidity in the insectary laboratory at the National Institute of Parasitic Diseases, Chinese Center for Disease Control and Prevention. Adult mosquitoes were maintained in screened cages and provided constant access to water and glucose-soaked sponges.

Male and female adults were identified morphologically and collected after emerging 3 to 4 days. All samples were flash-frozen in liquid nitrogen immediately following collection, and then stored at −80 °C until RNA isolation.

### 4.2. Phylogenetic Analysis of m6A Associated Proteins in Anopheles *spp.*

A total of 22 human m6A-associated proteins sequences (Table S5) were used as initial queries to search homologues in 22 *Anopheles* spp. genomes (https://vectorbase.org/vectorbase, accessed on 24 September 2021) with reciprocal Blast method, with an E-value cut-off < 0.05. The amino acid residues were visualized with Jalview Version 2 [61]. Sequences were aligned using MUSCLE (https://www.ebi.ac.uk/Tools/msa/muscle/, accessed on 26 September 2021) with the default parameters. Human and *Drosophila* were used as the out-group. These alignments were trimmed with TrimAI using the heuristic automated1 method [62]. Maximum likelihood (ML) analyses were performed using online IQ-TREE platform [63]. The best-fitting model was defined by IQ-TREE with Bayesian Information Criterion. The tree branches were tested with ultrafast bootstrapping (1000) and SH-like approximate likelihood ratio test (SH-aLRT, 1000 replicates). The final trees were visualized with TreeGraph2 [64]. The conserved domains were identified with NCBI Conserved Domain Search [65].

### 4.3. RNA Extraction and qPCR

Total RNA of male and female adult *Anopheles sinensis* was extracted separately using TRIzol reagent (Invitrogen, Carlsbad, CA, USA) following the manufacturer’s instructions. The RNA amount and purity of each sample were quantified using NanoDrop ND-1000 (NanoDrop, Wilmington, DE, USA). The integrity of the RNA was determined using the Agilent BioAnalyzer 2100 (Agilent Technologies, USA). Reverse transcription for qPCR was performed with PrimeScript RT master mix (Takara, Japan). Quantitative PCR was carried out using TB Green Premix Ex Taq II (Takara, Japan). The primer sequences specific to target genes were listed in Table S6. The cycling protocol was as follows: 95 °C for 30 s, followed by 35 cycles of 95 °C for 5 s, 60 °C for 10 s, and 72 °C for 30 s. A melting curve was generated by cooling the products to 65 °C and then heating to 95 °C at a rate of 0.1 °C/s while simultaneously measuring fluorescence. Samples were run in triplicate for technical repeats and triplicate for biological repeats. Relative enrichment levels were determined by comparison with *An. sinensis* 18 s rRNA [66], using the 2−ΔΔCt method.

### 4.4. Analysis of RNA Modification by Quantitative Mass Spectrometry

The Poly (A) RNA was purified from 200 μg total RNA using one round Dynabeads Oligo (dT) (Thermo Fisher, Carlsbad, CA, USA) purification. The mRNA samples (1 μg) were denatured at 95 °C for 5 min, digested by S1 nuclease (1 U), alkaline phosphatase (0.1 U), and phosphodiesterase (0.01 U) at 37 °C for 2 h. After RNA was digested into nucleosides completely, the mixture was extracted with chloroform to remove the enzymes. The aqueous layer in the mixture was collected for analysis with UPLC-ESI-MS/MS system (UPLC, ExionLC™ AD; MS, Applied Biosystems 6500 Triple Quadrupole) on the Metware platform (http://www.metware.cn/, accessed on 14 May 2021). The ribonucleosides in the hydrolyzed RNA samples run through Waters ACQUITY UPLC HSS T3 C18 (1.8 µm, 2.1 mm × 100 mm) in mobile phase buffer A (water with 2 mM NH4HCO3) and buffer B (Methanol with 2 mM NH4HCO3) at 0.30 mL/min, 40 °C. The elution profile was 95:5 (*V*/*V*) at 0 min, 95:5 at 1 min, 5:95 at 9 min, 5:95 at 11 min, 95:5 at 11.1 min, 95:5 at 14 min. The effluent was connected to an ESI-triple quadrupole-linear ion trap (QTRAP)-MS, equipped with an ESI Turbo Ion-Spray interface, operating in both positive and negative ion modes and controlled by Analyst 1.6.3 software (AB Sciex) under following parameters: ion source and turbo spray; source temperature of 550 °C; ion spray voltage (IS) of 5500 V. Specific multiple reaction monitoring (MRM) transitions (*m*/*z* 282.1 -> 150.1) were monitored for m6A nucleosides.

### 4.5. Mapping m6A Modifications in the Transcriptome

mRNA-seq and MeRIP-seq libraries. The Poly (A) RNA was purified from 50 μg total RNA using Dynabeads Oligo (dT) (Thermo Fisher, Carlsbad CA, USA) using two rounds of purification. Then the poly(A) RNA was fragmented into small pieces using Magnesium RNA Fragmentation Module (NEB, Ipswich, MA, USA) at 86 °C for 7 min. Cleaved RNA fragments were incubated with m6A-specific antibody (Synaptic Systems GmbH, Goettingen, Lower Saxony, Germany) for 2 h at 4 °C in IP buffer (50 mM Tris-HCl, 750 mM NaCl, and 0.5% Igepal CA-630). Both IP RNA and total Poly (A) RNA (input) were reverse-transcribed to create the cDNA by SuperScript™ II Reverse Transcriptase (Invitrogen, Carlsbad, CA, USA), which were next used to synthesize U-labeled second-stranded DNAs with *E. coli* DNA polymerase I (NEB, Ipswich, MA, USA), RNase H (NEB, Ipswich, MA, USA) and dUTP Solution (Thermo Fisher, Carlsbad, CA, USA). An A-base was then added to the blunt ends of each strand and ligate to the T-base overhang of adapters. The size selection for ligated fragments was performed with AMPureXP beads (NEB, Ipswich, MA, USA). After digesting the U-labeled second-stranded DNAs with UDG enzyme (NEB, Ipswich, MA, USA), the ligated products were amplified with PCR by the following conditions: initial denaturation at 95 °C for 3 min, 8 cycles of denaturation at 98 °C for 15 s, annealing at 60 °C for 15 s, extension at 72 °C for 30 s, final extension at 72 °C for 5 min. The cDNA libraries were sequenced at LC-Bio (Hangzhou, Zhejiang, China) using an illumina Novaseq™ 6000 instrument. Libraries from the samples were sequenced in 2 × 150 bp paired-end sequencing (PE150) mode, with average insertions length of 300 ± 50 bp. Raw data of RNA-seq and m6A-seq have been uploaded to GEO database (accession number GSE193379).

Read processing. Fastq files for male and female mosquitoes were trimmed using Fastp software [67] to remove the reads that contained adaptor contamination, low-quality bases, and undetermined bases with default parameter. RNA-seq data from control mRNA and m6A mRNA were mapped to *Anopheles sinensis* reference genome sequence (Version: v49) using HISAT2 package [68].

Peak calling and motif analysis. Mapped reads of IP and input cDNA libraries from male and female mosquitoes were used to identify peaks by R package exomePeak [69]. The m6A peaks were annotated using R package ChIPseeker [70] and visualized using IGV software [71]. The de novo and known motif predicting were performed using MEME [72] and HOMER [73] to locate the peaks of the motif.

Expression level of mRNA and analysis. The read counts per gene were then normalized to obtain FPKM values (total exon fragments /mapped reads (millions) × exon length (kB)) using StringTie [74]. Differentially expressed genes and differences in abundance of m6A peaks between male and female mosquitoes were calculated as fold change (FC), with absolute log2 FC ≥ 1 and *p* < 0.05 by R package edgeR [75]. Gene ontology (GO) and KEGG enrichment were carried out with online tools of LC-BIO company (https://www.omicstudio.cn/index, accessed on 22 October 2021).

## 5. Conclusions

*An. sinensis* is the vector for several parasites and viruses threatening human health, and novel tools targeting mosquito physiological developments and physical behaviors in the life cycle are urgently needed for vector control. In the present study, the profile of the transcriptome-wide m6A in male and female *An. sinensis* is characterized for the first time, and it is confirmed that m6A is a highly conserved modification in mosquitoes but with distinct characteristics compared to other species, including mammals and yeast. Moreover, unique distribution patterns of m6A in *An. sinensis* are associated with mosquito sex-specific pathways, especially in regulating spermatogenesis and the sperm tail formation of *An. sinensis*. This evidence paves the way for revealing the detailed regulation mechanism in mosquito physiological development and is a starting roadmap for uncovering m6A functions that may affect/control mosquito development in future, contributing to the control of this disease-transmitting vector.

## Figures and Tables

**Table 1:** The regulation patterns of m6A methylation and gene expression in female and male *An.* *sinensis*.

Gene_ID	Description	m6A					Gene Expression			
		Position	Diff. log2 (fc)	Diff. *p*_Value	Regulation	Position	log2 (fc)	*p*_Value	Regulation	Validated by qPCR
ASIS004369	testis-specific serine/threonine-protein kinase 2	5′ UTR	3.28	0.00	up	5′ UTR	inf	0.00	up	up
ASIS019512	dynein regulatory complex protein 11	3′ UTR	2.35	0.00	up	3′ UTR	7.45	0.00	up	
ASIS020833	Tctex1 domain containing 1	other Exon	1.37	0.04	up	other Exon	7.28	0.00	up	up
ASIS001560	cilia- and flagella-associated protein	other Exon	3.93	0.00	up	other Exon	6.73	0.00	up	up
ASIS008463	arginine kinase	other Exon	1.89	0.01	up	other Exon	6.25	0.00	up	up
		other Exon	1.54	0.03	up	other Exon	6.25	0.00	up	up
		3′ UTR	1.02	0.00	up	3′ UTR	6.25	0.00	up	up
ASIS009175	lethal (2) 41Ab	1st Exon	2.27	0.00	up	1st Exon	6.16	0.00	up	up
ASIS021651	male fertility factor kl3	other Exon	1.44	0.03	up	other Exon	5.79	0.00	up	
		other Exon	1.35	0.04	up	other Exon	5.79	0.00	up	
		other Exon	1.21	0.01	up	other Exon	5.79	0.00	up	
ASIS021865	tektin-3	5′ UTR	2.58	0.00	up	5′ UTR	5.76	0.00	up	up
ASIS012597	kelch-like protein 10	3′ UTR	1.29	0.05	up	3′ UTR	4.96	0.00	up	up
ASIS012445	unkown	5′ UTR	3.68	0.00	up	5′ UTR	4.40	0.00	up	
ASIS023324	unkown	other Exon	2.51	0.04	up	other Exon	3.70	0.00	up	up
ASIS015071	unkown	3′ UTR	1.23	0.00	up	3′ UTR	2.05	0.00	up	
ASIS016262	beta amyloid protein precursor-like	3′ UTR	1.64	0.00	up	3′ UTR	1.80	0.00	up	
ASIS018187	CNMamide receptor	1st Exon	3.22	0.01	up	1st Exon	1.72	0.00	up	
ASIS023203	unkown	other Exon	3.40	0.03	up	other Exon	1.72	0.01	up	
ASIS007683	unkown	other Exon	1.63	0.00	up	other Exon	1.71	0.00	up	
ASIS004699	unkown	1st Exon	3.06	0.03	up	1st Exon	1.39	0.00	up	
ASIS000944	Isocitte dehydrogenase [NAD] subunit, mitochondrial	3′ UTR	1.01	0.00	up	3′ UTR	1.33	0.00	up	
ASIS019305	fizzy related protein	1st Exon	1.31	0.01	up	1st Exon	1.28	0.00	up	
ASIS013151	unkown	1st Exon	1.05	0.01	up	1st Exon	1.17	0.00	up	
ASIS022379	CLIP-domain serine protease	other Exon	1.29	0.03	up	other Exon	1.13	0.01	up	
ASIS014174	unkown	1st Exon	1.13	0.01	up	1st Exon	1.00	0.04	up	
ASIS015991	DUF4746 domain containing protein	other Exon	−1.01	0.05	down	other Exon	6.93	0.00	up	up
ASIS017518	F-box domain-containing protein	1st Exon	−1.36	0.00	down	1st Exon	2.48	0.00	up	
ASIS022721	unkown	other Exon	−1.18	0.00	down	other Exon	2.17	0.00	up	
ASIS020661	unkown	other Exon	−1.39	0.01	down	other Exon	2.06	0.00	up	
ASIS022861	unkown	1st Exon	−1.40	0.00	down	1st Exon	1.79	0.00	up	
ASIS002904	syntaxin 1A	1st Exon	−5.84	0.00	down	1st Exon	1.68	0.00	up	up
ASIS003221	solute carrier family 25 member 45	3′ UTR	−1.62	0.02	down	3′ UTR	1.58	0.00	up	
ASIS022100	BTB/POZ domain-containing protein KCTD16	1st Exon	−1.78	0.00	down	1st Exon	1.35	0.00	up	
ASIS009765	microtubule-associated protein	other Exon	−2.02	0.02	down	other Exon	1.06	0.00	up	
ASIS007070	anaphase-promoting complex subunit 4	other Exon	1.72	0.00	up	other Exon	−1.23	0.00	down	
ASIS004428	very low-density lipoprotein receptor	1st Exon	1.07	0.00	up	1st Exon	−1.70	0.02	down	
ASIS011700	unkown	3′ UTR	1.15	0.00	up	3′ UTR	−2.12	0.00	down	
ASIS022740	ARF GTPase-activating protein GIT2	1st Exon	3.62	0.00	up	1st Exon	−2.43	0.00	down	
ASIS001538	unkown	other Exon	3.18	0.01	up	other Exon	−6.53	0.00	down	down
ASIS019082	unkown	3′ UTR	1.33	0.01	up	3′ UTR	−9.07	0.00	down	down
ASIS001385	multiple PDZ domain protein	1st Exon	−3.83	0.02	down	1st Exon	−2.32	0.00	down	down
ASIS006653	Niemann-Pick C1 protein	1st Exon	−2.94	0.00	down	1st Exon	−5.05	0.00	down	
ASIS000668	epidermal retinal dehydrogenase	3′ UTR	−2.67	0.02	down	3′ UTR	−4.65	0.00	down	down
ASIS017710	LDL receptor related protein 4,	5′ UTR	−2.04	0.00	down	5′ UTR	−2.03	0.00	down	
ASIS002714	dim gamma-tubulin 5	1st Exon	−1.70	0.03	down	1st Exon	−2.64	0.00	down	
ASIS004639	salivary gland protein 1-like	1st Exon	−1.47	0.00	down	1st Exon	−10.5	0.00	down	down
ASIS008153	chromosome transmission fidelity protein 18	other Exon	−1.41	0.04	down	other Exon	−1.26	0.00	down	
ASIS010595	Laminin-like protein epi-1	1st Exon	−1.37	0.01	down	1st Exon	−2.99	0.00	down	
ASIS019764	origin recognition complex subunit	5′ UTR	−1.25	0.01	down	5′ UTR	−1.72	0.00	down	
ASIS007143	chromosome transmission fidelity protein	1st Exon	−1.23	0.00	down	1st Exon	−3.20	0.00	down	
ASIS001584	mini chromosome maintenance-related protein	other Exon	−1.23	0.03	down	other Exon	−1.16	0.00	down	
ASIS005004	ANK_REP_REGION domain-containing protein	1st Exon	−1.18	0.04	down	1st Exon	−2.46	0.00	down	
ASIS013445	NudC domain containing 1	1st Exon	−1.16	0.00	down	1st Exon	−1.77	0.00	down	
ASIS018863	poly(A) RNA polymerase GLD2	other Exon	−1.13	0.00	down	other Exon	−2.70	0.00	down	
ASIS020303	glutaminyl-tRNA synthetase	3′ UTR	−1.07	0.00	down	3′ UTR	−1.11	0.00	down	
ASIS019128	yemanuclein	3′ UTR	−1.04	0.00	down	3′ UTR	−1.38	0.00	down	
ASIS014406	Ovo protein	3′ UTR	−1.03	0.00	down	3′ UTR	−1.87	0.00	down	

## Data Availability

Raw data of RNA-seq and m6A-seq have been uploaded to GEO database (accession number GSE193379).

## Supplementary Materials

The following supporting information can be downloaded at: https://www.mdpi.com/article/10.3390/ijms23094630/s1.

## References

[1] Feng J., Tu H., Zhang L., Xia Z., Zhou S. (2020). Imported Malaria Cases—China, 2012–2018. China CDC Wkly..

[2] Feng X., Zhang S., Huang F., Zhang L., Feng J., Xia Z., Zhou H., Hu W., Zhou S. (2017). Biology, Bionomics and Molecular Biology of *Anopheles sinensis* Wiedemann 1828 (Diptera: Culicidae), Main Malaria Vector in China. Front. Microbiol..

[3] Zhang S., Guo S., Feng X., Afelt A., Frutos R., Zhou S., Manguin S. (2017). *Anopheles* Vectors in Mainland China While Approaching Malaria Elimination. Trends Parasitol..

[4] Liu C.F., Qian H.L., Gu Z.C., Chang X., Wu Z.Y., Chen F.Q., Chen Y.Z. (1984). The role of *Anopheles lesteri anthropothagus* in malaria transmission in Jianghuai region, Anhui. J. Parasitol. Parasit. Dis..

[5] Sun Y., Yu D., Chen J., Li X., Wang B., Wang Z., Mao L., Yao W. (2017). Two individual incidences of vivax malaria in Dandong municipality of Liaoning province. Chin. J. Public Health.

[6] Zhang L., Feng J., Xia Z., Zhou S. (2020). Epidemiological characteristics of malaria and progress on its elimination in China in 2019. Chin. J. Parasitol. Parasit. Dis..

[7] Wang D., Li S., Cheng Z., Xiao N., Cotter C., Hwang J., Li X., Yin S., Wang J., Bai L. (2015). Transmission Risk from Imported *Plasmodium vivax* Malaria in the China-Myanmar Border Region. Emerg. Infect. Dis..

[8] Zhang S., Cheng F., Webber R. (1994). A successful control programme for lymphatic filariasis in Hubei, China. Trans. R. Soc. Trop. Med. Hyg..

[9] Manguin S., Bangs M.J., Pothikasikorn J., Chareonviriyaphap T. (2010). Review on global co-transmission of human *Plasmodium* species and *Wuchereria bancrofti* by *Anopheles* mosquitoes. Infect. Genet. Evol..

[10] Oliveira A., Strathe E., Etcheverry L., Cohnstaedt L.W., McVey D.S., Piaggio J., Cernicchiaro N. (2018). Assessment of data on vector and host competence for *Japanese encephalitis virus*: A systematic review of the literature. Prev. Vet. Med..

[11] Barua S., Hoque M.M., Kelly P.J., Poudel A., Adekanmbi F., Kalalah A., Yang Y., Wang C. (2020). First report of *Rickettsia felis* in mosquitoes, USA. Emerg. Microbes. Infect..

[12] Wang S., Dos-Santos A., Huang W., Liu K.C., Oshaghi M.A., Wei G., Agre P., Jacobs-Lorena M. (2017). Driving mosquito refractoriness to *Plasmodium falciparum* with engineered symbiotic bacteria. Science.

[13] Glastad K.M., Hunt B.G., Goodisman M. (2019). Epigenetics in Insects: Genome Regulation and the Generation of Phenotypic Diversity. Annu. Rev. Entomol..

[14] Haussmann I.U., Bodi Z., Sanchez-Moran E., Mongan N.P., Archer N., Fray R.G., Soller M. (2016). m6A potentiates Sxl alternative pre-mRNA splicing for robust *Drosophila* sex determination. Nature.

[15] Kan L., Grozhik A.V., Vedanayagam J., Patil D.P., Pang N., Lim K.S., Huang Y.C., Joseph B., Lin C.J., Despic V. (2017). The m6A pathway facilitates sex determination in *Drosophila*. Nat. Commun..

[16] Lence T., Akhtar J., Bayer M., Schmid K., Spindler L., Ho C.H., Kreim N., Andrade-Navarro M.A., Poeck B., Helm M. (2016). m6A modulates neuronal functions and sex determination in *Drosophila*. Nature.

[17] Worpenberg L., Paolantoni C., Longhi S., Mulorz M.M., Lence T., Wessels H.H., Dassi E., Aiello G., Sutandy F., Scheibe M. (2021). Ythdf is a N6-methyladenosine reader that modulates Fmr1 target mRNA selection and restricts axonal growth in *Drosophila*. EMBO J..

[18] Guo J., Tang H.W., Li J., Perrimon N., Yan D. (2018). Xio is a component of the *Drosophila* sex determination pathway and RNA N6-methyladenosine methyltransferase complex. Proc. Natl. Acad. Sci. USA.

[19] Wang M., Xiao Y., Li Y., Wang X., Qi S., Wang Y., Zhao L., Wang K., Peng W., Luo G.Z. (2021). RNA m6A Modification Functions in Larval Development and Caste Differentiation in Honeybee (*Apis mellifera*). Cell Rep..

[20] Rockwell A.L., Hongay C.F. (2020). Dm Ime4 depletion affects permeability barrier and Chic function in *Drosophila* spermatogenesis. Mech. Dev..

[21] Yang X., Wei X., Yang J., Du T., Yin C., Fu B., Huang M., Liang J., Gong P., Liu S. (2021). Epitranscriptomic regulation of insecticide resistance. Sci. Adv..

[22] Jiang T., Li J., Qian P., Xue P., Xu J., Chen Y., Zhu J., Tang S., Zhao Q., Qian H. (2019). The role of N6-methyladenosine modification on diapause in silkworm (*Bombyx mori*) strains that exhibit different voltinism. Mol. Reprod. Dev..

[23] Leismann J., Spagnuolo M., Pradhan M., Wacheul L., Vu M.A., Musheev M., Mier P., Andrade-Navarro M.A., Graille M., Niehrs C. (2020). The 18S ribosomal RNA m6 A methyltransferase Mettl5 is required for normal walking behavior in *Drosophila*. EMBO Rep..

[24] Zaccara S., Ries R.J., Jaffrey S.R. (2019). Reading, writing and erasing mRNA methylation. Nat. Rev. Mol. Cell Biol..

[25] Dominissini D., Moshitch-Moshkovitz S., Schwartz S., Salmon-Divon M., Ungar L., Osenberg S., Cesarkas K., Jacob-Hirsch J., Amariglio N., Kupiec M. (2012). Topology of the human and mouse m6A RNA methylomes revealed by m6A-seq. Nature.

[26] Luo G.Z., MacQueen A., Zheng G., Duan H., Dore L.C., Lu Z., Liu J., Chen K., Jia G., Bergelson J. (2014). Unique features of the m6A methylome in *Arabidopsis thaliana*. Nat. Commun..

[27] Schwartz S., Agarwala S.D., Mumbach M.R., Jovanovic M., Mertins P., Shishkin A., Tabach Y., Mikkelsen T.S., Satija R., Ruvkun G. (2013). High-resolution mapping reveals a conserved, widespread, dynamic mRNA methylation program in yeast meiosis. Cell.

[28] Jiang X., Liu B., Nie Z., Duan L., Xiong Q., Jin Z., Yang C., Chen Y. (2021). The role of m6A modification in the biological functions and diseases. Signal. Transduct. Target Ther..

[29] Liu N., Dai Q., Zheng G., He C., Parisien M., Pan T. (2015). N(6)-methyladenosine-dependent RNA structural switches regulate RNA-protein interactions. Nature.

[30] Lence T., Soller M., Roignant J.Y. (2017). A fly view on the roles and mechanisms of the m6A mRNA modification and its players. RNA Biol..

[31] Zhao X., Yang Y., Sun B.F., Shi Y., Yang X., Xiao W., Hao Y.J., Ping X.L., Chen Y.S., Wang W.J. (2014). FTO-dependent demethylation of N6-methyladenosine regulates mRNA splicing and is required for adipogenesis. Cell Res..

[32] Tang C., Klukovich R., Peng H., Wang Z., Yu T., Zhang Y., Zheng H., Klungland A., Yan W. (2018). ALKBH5-dependent m6A demethylation controls splicing and stability of long 3’-UTR mRNAs in male germ cells. Proc. Natl. Acad. Sci. USA.

[33] Shao X.L., He S.Y., Zhuang X.Y., Fan Y., Li Y.H., Yao Y.G. (2014). mRNA expression and DNA methylation in three key genes involved in caste differentiation in female honeybees (*Apis mellifera*). Zool. Res..

[34] Krafsur E.S., Jones J.C. (1967). Spermiogenesis in *Aedes aegypti* (L.). Cytologia.

[35] Gui Y., Yuan S. (2021). Epigenetic regulations in mammalian spermatogenesis: RNA-m6A modification and beyond. Cell. Mol. Life Sci..

[36] Zhou Y., Kong Y., Fan W., Tao T., Xiao Q., Li N., Zhu X. (2020). Principles of RNA methylation and their implications for biology and medicine. Biomed. Pharmacother..

[37] Xu K., Yang Y., Feng G.H., Sun B.F., Chen J.Q., Li Y.F., Chen Y.S., Zhang X.X., Wang C.X., Jiang L.Y. (2017). Mettl3-mediated m6A regulates spermatogonial differentiation and meiosis initiation. Cell Res..

[38] Lin Z., Hsu P.J., Xing X., Fang J., Lu Z., Zou Q., Zhang K.J., Zhang X., Zhou Y., Zhang T. (2017). Mettl3-/Mettl14-mediated mRNA N6-methyladenosine modulates murine spermatogenesis. Cell Res..

[39] Hsu P.J., Zhu Y., Ma H., Guo Y., Shi X., Liu Y., Qi M., Lu Z., Shi H., Wang J. (2017). Ythdc2 is an N6-methyladenosine binding protein that regulates mammalian spermatogenesis. Cell Res..

[40] Clements A.N., Potter S.A. (1967). The fine structure of the spermathecae and their ducts in the mosquito *Aedes aegypti*. J. Insect Physiol..

[41] Degner E.C., Harrington L.C. (2016). A mosquito sperm’s journey from male ejaculate to egg: Mechanisms, molecules, and methods for exploration. Mol. Reprod. Dev..

[42] Degner E.C., Ahmed-Braimah Y.H., Borziak K., Wolfner M.F., Harrington L.C., Dorus S. (2019). Proteins, Transcripts, and Genetic Architecture of Seminal Fluid and Sperm in the Mosquito *Aedes aegypti*. Mol. Cell. Proteom..

[43] Wang S., Zheng H., Esaki Y., Kelly F., Yan W. (2006). Cullin3 is a KLHL10-interacting protein preferentially expressed during late spermiogenesis. Biol. Reprod..

[44] Gantz V.M., Jasinskiene N., Tatarenkova O., Fazekas A., Macias V.M., Bier E., James A.A. (2015). Highly efficient Cas9-mediated gene drive for population modification of the malaria vector mosquito *Anopheles stephensi*. Proc. Natl. Acad. Sci. USA.

[45] Hammond A., Galizi R., Kyrou K., Simoni A., Siniscalchi C., Katsanos D., Gribble M., Baker D., Marois E., Russell S. (2016). A CRISPR-Cas9 gene drive system targeting female reproduction in the malaria mosquito vector *Anopheles gambiae*. Nat. Biotechnol..

[46] Adelman Z.N., Tu Z. (2016). Control of Mosquito-Borne Infectious Diseases: Sex and Gene Drive. Trends Parasitol..

[47] Simoni A., Hammond A.M., Beaghton A.K., Galizi R., Taxiarchi C., Kyrou K., Meacci D., Gribble M., Morselli G., Burt A. (2020). A male-biased sex-distorter gene drive for the human malaria vector *Anopheles gambiae*. Nat. Biotechnol..

[48] Krzywinska E., Dennison N.J., Lycett G.J., Krzywinski J. (2016). A maleness gene in the malaria mosquito *Anopheles gambiae*. Science.

[49] Sinkins S.P. (2016). SEX DETERMINATION. Yob makes mosquitoes male. Science.

[50] Cator L.J., Wyer C., Harrington L.C. (2021). Mosquito Sexual Selection and Reproductive Control Programs. Trends Parasitol..

[51] Burki F., Roger A.J., Brown M.W., Simpson A. (2020). The New Tree of Eukaryotes. Trends Ecol. Evol..

[52] Li B., Wang X., Li Z., Lu C., Zhang Q., Chang L., Li W., Cheng T., Xia Q., Zhao P. (2019). Transcriptome-wide analysis of N6-methyladenosine uncovers its regulatory role in gene expression in the lepidopteran *Bombyx mori*. Insect Mol. Biol..

[53] Su T., Fu L., Kuang L., Chen D., Zhang G., Shen Q., Wu D. (2022). Transcriptome-wide m6A methylation profile reveals regulatory networks in roots of barley under cadmium stress. J. Hazard. Mater..

[54] Yang Y., Huang W., Huang J.T., Shen F., Xiong J., Yuan E.F., Qin S.S., Zhang M., Feng Y.Q., Yuan B.F. (2016). Increased N6-methyladenosine in Human Sperm RNA as a Risk Factor for Asthenozoospermia. Sci. Rep..

[55] Xia H., Zhong C., Wu X., Chen J., Tao B., Xia X., Shi M., Zhu Z., Trudeau V.L., Hu W. (2018). Mettl3 Mutation Disrupts Gamete Maturation and Reduces Fertility in Zebrafish. Genetics.

[56] Zheng G., Dahl J.A., Niu Y., Fedorcsak P., Huang C.M., Li C.J., Vågbø C.B., Shi Y., Wang W.L., Song S.H. (2013). ALKBH5 is a mammalian RNA demethylase that impacts RNA metabolism and mouse fertility. Mol. Cell.

[57] Zheng G., Dahl J.A., Niu Y., Fu Y., Klungland A., Yang Y.G., He C. (2013). Sprouts of RNA epigenetics: The discovery of mammalian RNA demethylases. RNA Biol..

[58] Jain D., Puno M.R., Meydan C., Lailler N., Mason C.E., Lima C.D., Anderson K.V., Keeney S. (2018). Ketu mutant mice uncover an essential meiotic function for the ancient RNA helicase YTHDC2. eLife.

[59] Huang T., Liu Z., Zheng Y., Feng T., Gao Q., Zeng W. (2020). YTHDF2 promotes spermagonial adhesion through modulating MMPs decay via m6A/mRNA pathway. Cell Death Dis..

[60] Lin Z., Tong M.H. (2019). m6A mRNA modification regulates mammalian spermatogenesis. Biochim. Biophys. Acta Gene Regul. Mech..

[61] Waterhouse A.M., Procter J.B., Martin D.M.A., Clamp M., Barton G.J. (2009). Jalview Version 2—A multiple sequence alignment editor and analysis workbench. Bioinformatics.

[62] Capella-Gutiérrez S., Silla-Martínez J.M., Gabaldón T. (2009). trimAl: A tool for automated alignment trimming in large-scale phylogenetic analyses. Bioinformatics.

[63] Trifinopoulos J., Nguyen L.T., von Haeseler A., Minh B.Q. (2016). W-IQ-TREE: A fast online phylogenetic tool for maximum likelihood analysis. Nucleic Acids Res..

[64] Stöver B.C., Müller K.F. (2010). TreeGraph 2: Combining and visualizing evidence from different phylogenetic analyses. BMC Bioinformat..

[65] Lu S., Wang J., Chitsaz F., Derbyshire M.K., Geer R.C., Gonzales N.R., Gwadz M., Hurwitz D.I., Marchler G.H., Song J.S. (2020). CDD/SPARCLE: The conserved domain database in 2020. Nucleic Acids Res..

[66] Zhu G., Zhong D., Cao J., Zhou H., Li J., Liu Y., Bai L., Xu S., Wang M.H., Zhou G. (2014). Transcriptome profiling of pyrethroid resistant and susceptible mosquitoes in the malaria vector, *Anopheles sinensis*. BMC Genom..

[67] Chen S., Zhou Y., Chen Y., Gu J. (2018). Fastp: An ultra-fast all-in-one FASTQ preprocessor. Bioinformatics.

[68] Kim D., Langmead B., Salzberg S.L. (2015). HISAT: A fast spliced aligner with low memory requirements. Nat. Methods.

[69] Meng J., Lu Z., Liu H., Zhang L., Zhang S., Chen Y., Rao M.K., Huang Y. (2014). A protocol for RNA methylation differential analysis with MeRIP-Seq data and exomePeak R/Bioconductor package. Methods.

[70] Yu G., Wang L.G., He Q.Y. (2015). ChIPseeker: An R/Bioconductor package for ChIP peak annotation, comparison and visualization. Bioinformatics.

[71] Thorvaldsdóttir H., Robinson J.T., Mesirov J.P. (2013). Integrative Genomics Viewer (IGV): High-performance genomics data visualization and exploration. Brief Bioinform..

[72] Bailey T.L., Boden M., Buske F.A., Frith M., Grant C.E., Clementi L., Ren J., Li W.W., Noble W.S. (2009). MEME SUITE: Tools for motif discovery and searching. Nucleic Acids Res..

[73] Heinz S., Benner C., Spann N., Bertolino E., Lin Y.C., Laslo P., Cheng J.X., Murre C., Singh H., Glass C.K. (2010). Simple combinations of lineage-determining transcription factors prime cis-regulatory elements required for macrophage and B cell identities. Mol. Cell.

[74] Pertea M., Pertea G.M., Antonescu C.M., Chang T.C., Mendell J.T., Salzberg S.L. (2015). StringTie enables improved reconstruction of a transcriptome from RNA-seq reads. Nat. Biotechnol..

[75] Robinson M.D., McCarthy D.J., Smyth G.K. (2010). edgeR: A Bioconductor package for differential expression analysis of digital gene expression data. Bioinformatics.

