# Supplementary material for: Profiling of Transcriptome-Wide N6-Methyladenosine (m6A) Modifications and Identifying m6A Associated Regulation in Sperm Tail Formation in Anopheles sinensis
Source: Int J Mol Sci. 2022 Apr 22;23(9):4630. doi: 10.3390/ijms23094630 (PMC9101273; doi:10.3390/ijms23094630)
Supplement: Supplementary file 1 [file ijms-23-04630-s001.zip › supplementary file S1-revised.pdf]

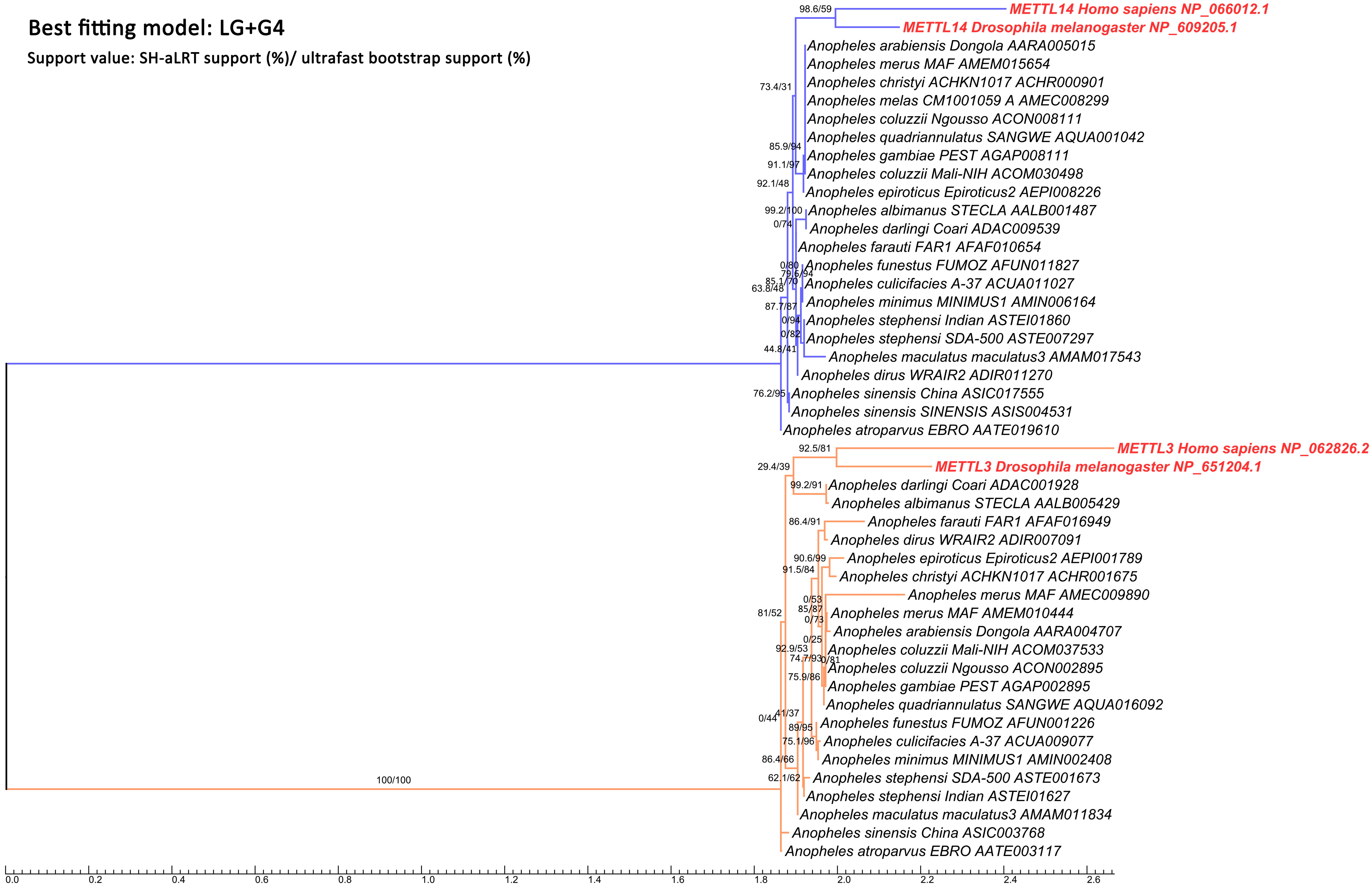

Best fitting model: LG+G4
Support value: SH-aLRT support (%)/ ultrafast bootstrap support (%)
METTL14 Homo sapiens NP_066012.1
METTL14 Drosophila melanogaster NP_609205.1
Anopheles arabiensis Dongola AARA005015
Anopheles merus MAF AMEM015654
Anopheles christyi ACHKN1017 ACHR000901
Anopheles melas CM1001059 A AMEC008299
Anopheles coluzzii Ngousso ACON008111
Anopheles quadriannulatus SANGWE AQUA001042
Anopheles gambiae PEST AGAP008111
Anopheles coluzzii Mali-NIH ACOM030498
Anopheles epiroticus Epiroticus2 AEPI008226
Anopheles albimanus STECLA AALB001487
Anopheles darlingi Coari ADAC009539
Anopheles farauti FAR1 AFAF010654
Anopheles funestus FUMOZ AFUN011827
Anopheles culicifacies A-37 ACUA011027
Anopheles minimus MINIMUS1 AMIN006164
Anopheles stephensi Indian ASTEI01860
Anopheles stephensi SDA-500 ASTE007297
Anopheles maculatus maculatus3 AMAM017543
Anopheles dirus WRAIR2 ADIR011270
Anopheles sinensis China ASIC017555
Anopheles sinensis SINENSIS ASIS004531
Anopheles atroparvus EBRO AATE019610
METTL3 Homo sapiens NP_062826.2
METTL3 Drosophila melanogaster NP_651204.1
Anopheles darlingi Coari ADAC001928
Anopheles albimanus STECLA AALB005429
Anopheles farauti FAR1 AFAF016949
Anopheles dirus WRAIR2 ADIR007091
Anopheles epiroticus Epiroticus2 AEPI001789
Anopheles christyi ACHKN1017 ACHR001675
Anopheles merus MAF AMEC009890
Anopheles merus MAF AMEM010444
Anopheles arabiensis Dongola AARA004707
Anopheles coluzzii Mali-NIH ACOM037533
Anopheles coluzzii Ngousso ACON002895
Anopheles gambiae PEST AGAP002895
Anopheles quadriannulatus SANGWE AQUA016092
Anopheles funestus FUMOZ AFUN001226
Anopheles culicifacies A-37 ACUA009077
Anopheles minimus MINIMUS1 AMIN002408
Anopheles stephensi SDA-500 ASTE001673
Anopheles stephensi Indian ASTEI01627
Anopheles maculatus maculatus3 AMAM011834
Anopheles sinensis China ASIC003768
Anopheles atroparvus EBRO AATE003117
100/100
0.0 0.2 0.4 0.6 0.8 1.0 1.2 1.4 1.6 1.8 2.0 2.2 2.4 2.6

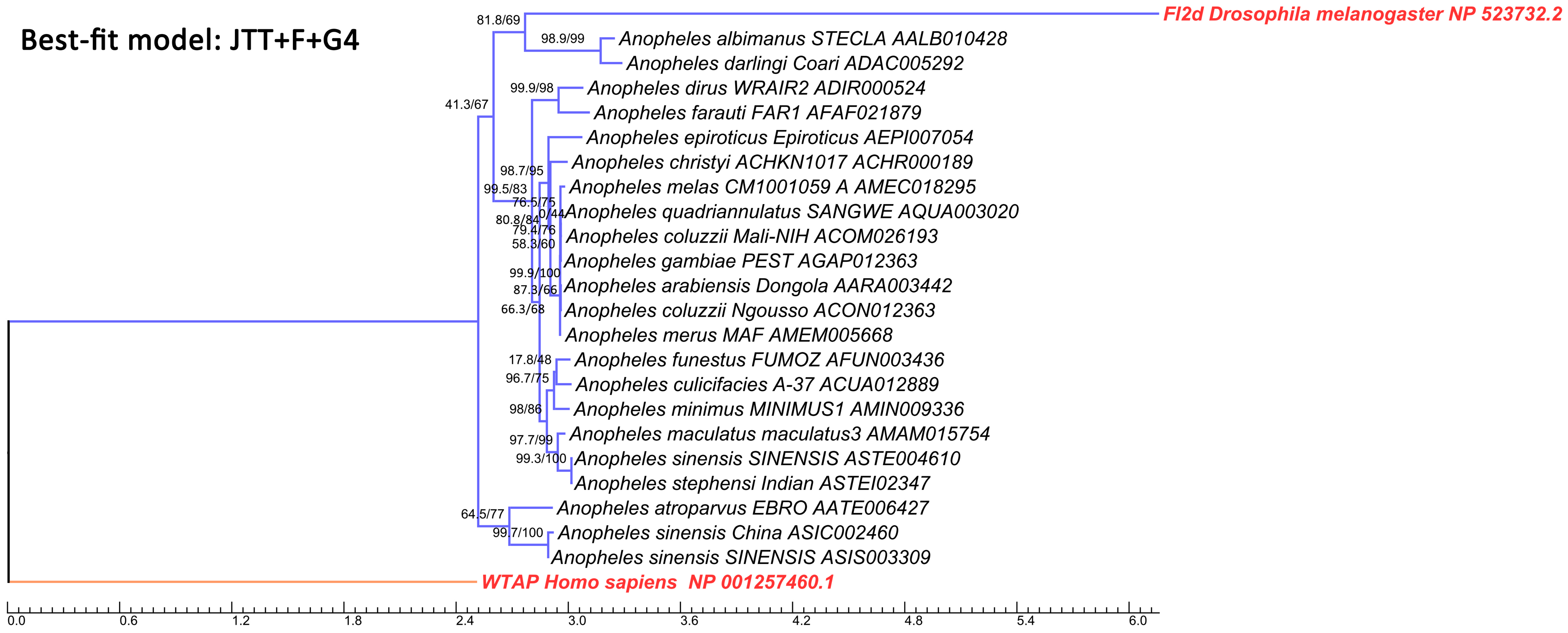

Best-fit model: JTT+F+G4
Fl2d Drosophila melanogaster NP 523732.2
Anopheles albimanus STECLA AALB010428
Anopheles darlingi Coari ADAC005292
Anopheles dirus WRAIR2 ADIR000524
Anopheles farauti FAR1 AFAF021879
Anopheles epiroticus Epiroticus AEPI007054
Anopheles christyi ACHKN1017 ACHR000189
Anopheles melas CM1001059 A AMEC018295
Anopheles quadriannulatus SANGWE AQUA003020
Anopheles coluzzii Mali-NIH ACOM026193
Anopheles gambiae PEST AGAP012363
Anopheles arabiensis Dongola AARA003442
Anopheles coluzzii Ngousso ACON012363
Anopheles merus MAF AMEM005668
Anopheles funestus FUMOZ AFUN003436
Anopheles culicifacies A-37 ACUA012889
Anopheles minimus MINIMUS1 AMIN009336
Anopheles maculatus maculatus3 AMAM015754
Anopheles sinensis SINENSIS ASTE004610
Anopheles stephensi Indian ASTEI02347
Anopheles atroparvus EBRO AATE006427
Anopheles sinensis China ASIC002460
Anopheles sinensis SINENSIS ASIS003309
WTAP Homo sapiens NP 001257460.1
81.8/69
98.9/99
99.9/98
41.3/67
98.7/95
99.5/83
76.5/75
80.8/84
0/44
79.4/76
58.3/60
99.9/100
87.3/66
66.3/68
17.8/48
96.7/75
98/86
97.7/99
99.3/100
64.5/77
99.7/100
0.0
0.6
1.2
1.8
2.4
3.0
3.6
4.2
4.8
5.4
6.0

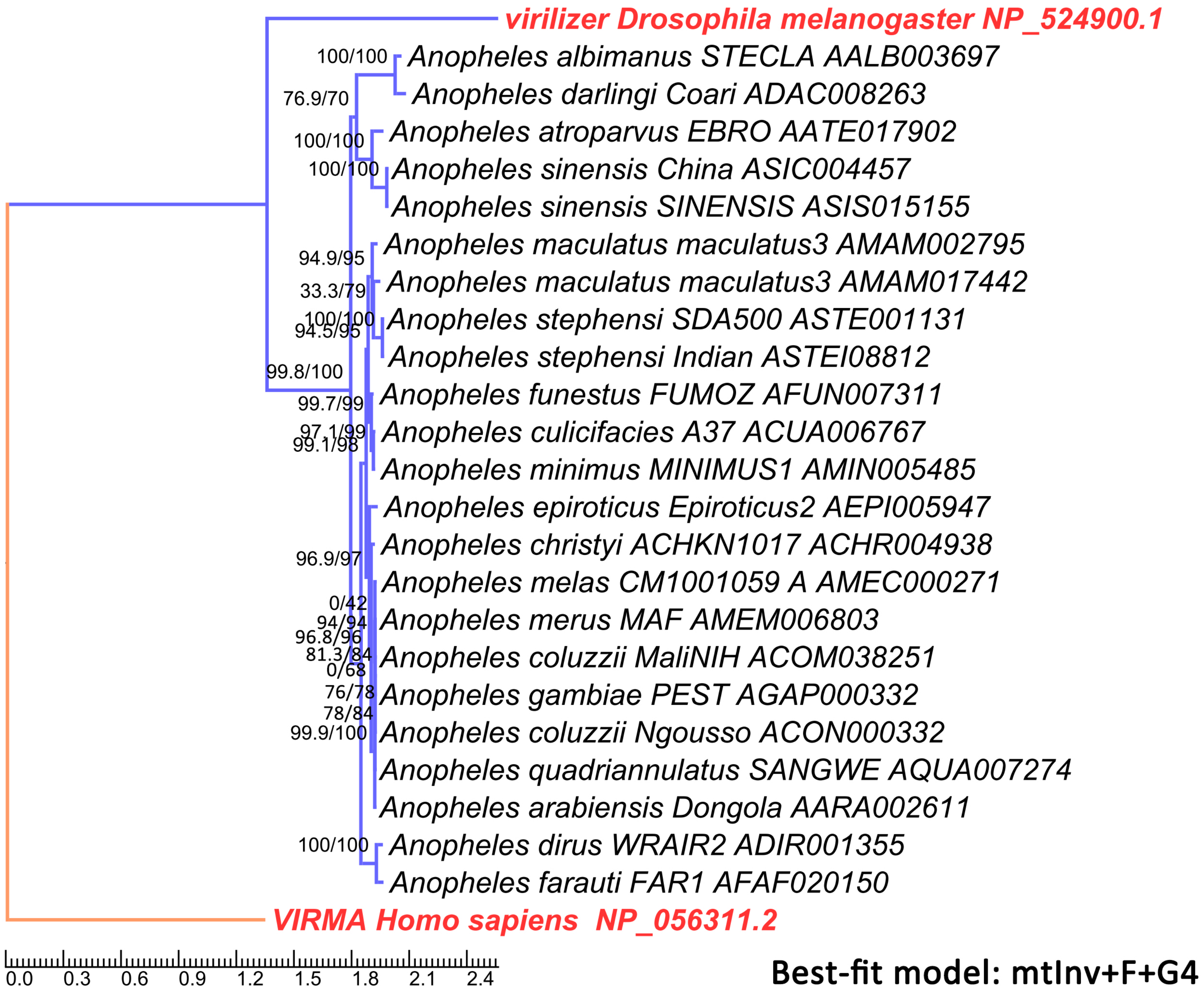
virilizer Drosophila melanogaster NP_524900.1
Anopheles albimanus STECLA AALB003697
Anopheles darlingi Coari ADAC008263
Anopheles atroparvus EBRO AATE017902
Anopheles sinensis China ASIC004457
Anopheles sinensis SINENSIS ASIS015155
Anopheles maculatus maculatus3 AMAM002795
Anopheles maculatus maculatus3 AMAM017442
Anopheles stephensi SDA500 ASTE001131
Anopheles stephensi Indian ASTEI08812
Anopheles funestus FUMOZ AFUN007311
Anopheles culicifacies A37 ACUA006767
Anopheles minimus MINIMUS1 AMIN005485
Anopheles epiroticus Epiroticus2 AEPI005947
Anopheles christyi ACHKN1017 ACHR004938
Anopheles melas CM1001059 A AMEC000271
Anopheles merus MAF AMEM006803
Anopheles coluzzii MaliNIH ACOM038251
Anopheles gambiae PEST AGAP000332
Anopheles coluzzii Ngousso ACON000332
Anopheles quadriannulatus SANGWE AQUA007274
Anopheles arabiensis Dongola AARA002611
Anopheles dirus WRAIR2 ADIR001355
Anopheles farauti FAR1 AFAF020150
VIRMA Homo sapiens NP_056311.2
100/100
76.9/70
100/100
100/100
94.9/95
33.3/79
100/100
94.5/95
99.8/100
99.7/99
97.1/99
99.1/98
96.9/97
0/42
94/94
96.8/96
81.3/84
0/68
76/78
78/84
99.9/100
100/100
0.0 0.3 0.6 0.9 1.2 1.5 1.8 2.1 2.4
Best-fit model: mtInv+F+G4

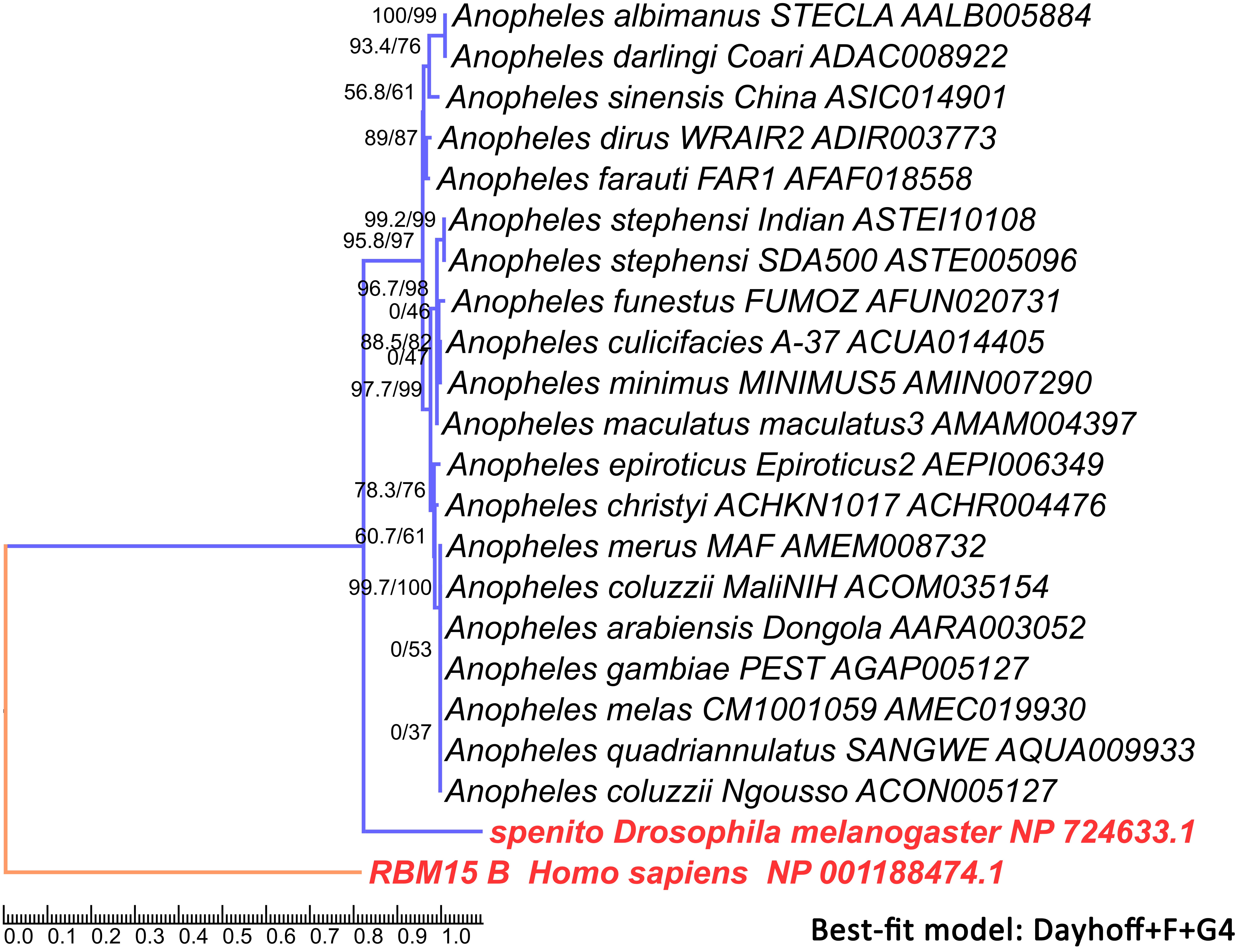

100/99
Anopheles albimanus STECLA AALB005884
93.4/76
Anopheles darlingi Coari ADAC008922
56.8/61
Anopheles sinensis China ASIC014901
89/87
Anopheles dirus WRAIR2 ADIR003773
Anopheles farauti FAR1 AFAF018558
99.2/99
Anopheles stephensi Indian ASTEI10108
95.8/97
Anopheles stephensi SDA500 ASTE005096
96.7/98
Anopheles funestus FUMOZ AFUN020731
0/46
88.5/82
Anopheles culicifacies A-37 ACUA014405
0/47
97.7/99
Anopheles minimus MINIMUS5 AMIN007290
Anopheles maculatus maculatus3 AMAM004397
Anopheles epiroticus Epiroticus2 AEPI006349
78.3/76
Anopheles christyi ACHKN1017 ACHR004476
60.7/61
Anopheles merus MAF AMEM008732
99.7/100
Anopheles coluzzii MaliNIH ACOM035154
Anopheles arabiensis Dongola AARA003052
0/53
Anopheles gambiae PEST AGAP005127
Anopheles melas CM1001059 AMEC019930
0/37
Anopheles quadriannulatus SANGWE AQUA009933
Anopheles coluzzii Ngousso ACON005127
spenito Drosophila melanogaster NP 724633.1
RBM15 B Homo sapiens NP 001188474.1
0.0 0.1 0.2 0.3 0.4 0.5 0.6 0.7 0.8 0.9 1.0
Best-fit model: Dayhoff+F+G4

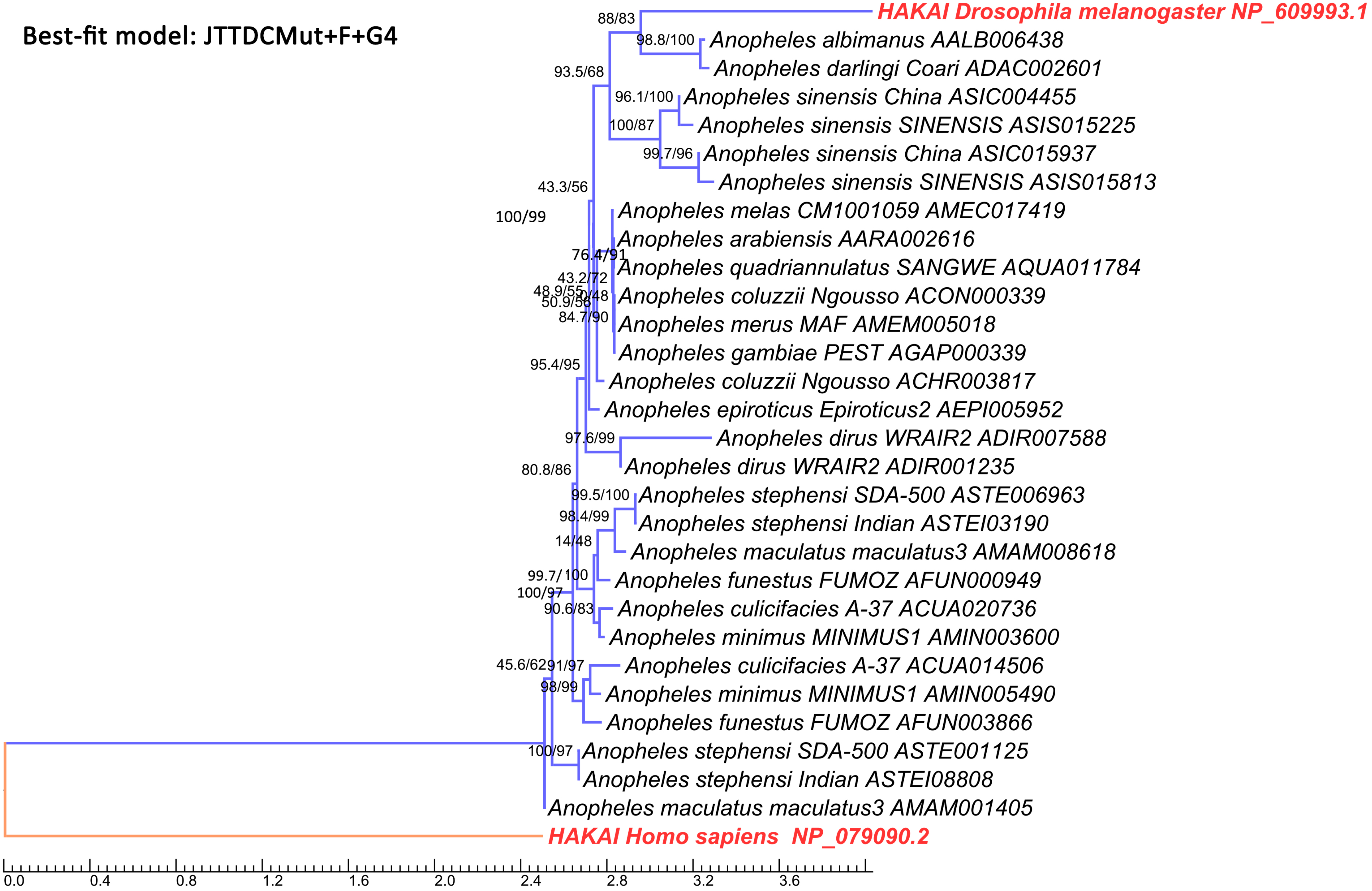

Best-fit model: JTTDCMut+F+G4
HAKAI Drosophila melanogaster NP_609993.1
Anopheles albimanus AALB006438
Anopheles darlingi Coari ADAC002601
Anopheles sinensis China ASIC004455
Anopheles sinensis SINENSIS ASIS015225
Anopheles sinensis China ASIC015937
Anopheles sinensis SINENSIS ASIS015813
Anopheles melas CM1001059 AMEC017419
Anopheles arabiensis AARA002616
Anopheles quadriannulatus SANGWE AQUA011784
Anopheles coluzzii Ngousso ACON000339
Anopheles merus MAF AMEM005018
Anopheles gambiae PEST AGAP000339
Anopheles coluzzii Ngousso ACHR003817
Anopheles epiroticus Epiroticus2 AEPI005952
Anopheles dirus WRAIR2 ADIR007588
Anopheles dirus WRAIR2 ADIR001235
Anopheles stephensi SDA-500 ASTE006963
Anopheles stephensi Indian ASTEI03190
Anopheles maculatus maculatus3 AMAM008618
Anopheles funestus FUMOZ AFUN000949
Anopheles culicifacies A-37 ACUA020736
Anopheles minimus MINIMUS1 AMIN003600
Anopheles culicifacies A-37 ACUA014506
Anopheles minimus MINIMUS1 AMIN005490
Anopheles funestus FUMOZ AFUN003866
Anopheles stephensi SDA-500 ASTE001125
Anopheles stephensi Indian ASTEI08808
Anopheles maculatus maculatus3 AMAM001405
HAKAI Homo sapiens NP_079090.2
88/83
98.8/100
93.5/68
96.1/100
100/87
99.7/96
43.3/56
100/99
76.4/91
43.2/72
48.9/55
50.9/58
84.7/90
95.4/95
97.6/99
80.8/86
99.5/100
98.4/99
14/48
99.7/100
100/97
90.6/83
45.6/62
91/97
98/99
100/97
0.0
0.4
0.8
1.2
1.6
2.0
2.4
2.8
3.2
3.6

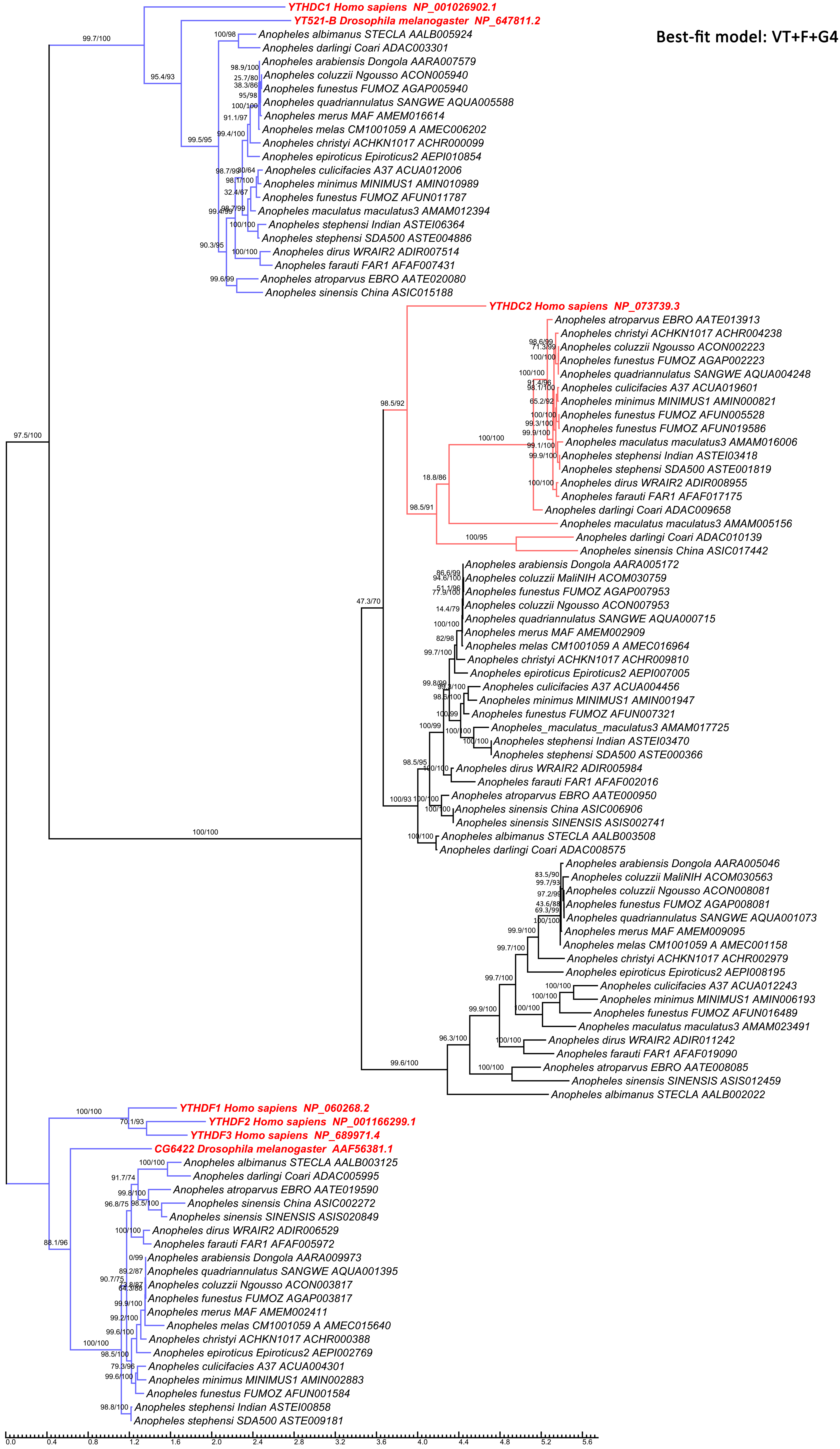
Best-fit model: VT+F+G4
YTHDC1 Homo sapiens NP_001026902.1
YT521-B Drosophila melanogaster NP_647811.2
Anopheles albimanus STECLA AALB005924
Anopheles darlingi Coari ADAC003301
Anopheles arabiensis Dongola AARA007579
Anopheles coluzzii Ngousso ACON005940
Anopheles funestus FUMOZ AGAP005940
Anopheles quadriannulatus SANGWE AQUA005588
Anopheles merus MAF AMEM016614
Anopheles melas CM1001059 A AMEC006202
Anopheles christyi ACHKN1017 ACHR000099
Anopheles epiroticus Epiroticus2 AEPI010854
Anopheles culicifacies A37 ACUA012006
Anopheles minimus MINIMUS1 AMIN010989
Anopheles funestus FUMOZ AFUN011787
Anopheles maculatus maculatus3 AMAM012394
Anopheles stephensi Indian ASTEI06364
Anopheles stephensi SDA500 ASTE004886
Anopheles dirus WRAIR2 ADIR007514
Anopheles farauti FAR1 AFAF007431
Anopheles atroparvus EBRO AATE020080
Anopheles sinensis China ASIC015188
YTHDC2 Homo sapiens NP_073739.3
Anopheles atroparvus EBRO AATE013913
Anopheles christyi ACHKN1017 ACHR004238
Anopheles coluzzii Ngousso ACON002223
Anopheles funestus FUMOZ AGAP002223
Anopheles quadriannulatus SANGWE AQUA004248
Anopheles culicifacies A37 ACUA019601
Anopheles minimus MINIMUS1 AMIN000821
Anopheles funestus FUMOZ AFUN005528
Anopheles funestus FUMOZ AFUN019586
Anopheles maculatus maculatus3 AMAM016006
Anopheles stephensi Indian ASTEI03418
Anopheles stephensi SDA500 ASTE001819
Anopheles dirus WRAIR2 ADIR008955
Anopheles farauti FAR1 AFAF017175
Anopheles darlingi Coari ADAC009658
Anopheles maculatus maculatus3 AMAM005156
Anopheles darlingi Coari ADAC010139
Anopheles sinensis China ASIC017442
Anopheles arabiensis Dongola AARA005172
Anopheles coluzzii MaliNIH ACOM030759
Anopheles funestus FUMOZ AGAP007953
Anopheles coluzzii Ngousso ACON007953
Anopheles quadriannulatus SANGWE AQUA000715
Anopheles merus MAF AMEM002909
Anopheles melas CM1001059 A AMEC016964
Anopheles christyi ACHKN1017 ACHR009810
Anopheles epiroticus Epiroticus2 AEPI007005
Anopheles culicifacies A37 ACUA004456
Anopheles minimus MINIMUS1 AMIN001947
Anopheles funestus FUMOZ AFUN007321
Anopheles_maculatus_maculatus3 AMAM017725
Anopheles stephensi Indian ASTEI03470
Anopheles stephensi SDA500 ASTE000366
Anopheles dirus WRAIR2 ADIR005984
Anopheles farauti FAR1 AFAF002016
Anopheles atroparvus EBRO AATE000950
Anopheles sinensis China ASIC006906
Anopheles sinensis SINENSIS ASIS002741
Anopheles albimanus STECLA AALB003508
Anopheles darlingi Coari ADAC008575
Anopheles arabiensis Dongola AARA005046
Anopheles coluzzii MaliNIH ACOM030563
Anopheles coluzzii Ngousso ACON008081
Anopheles funestus FUMOZ AGAP008081
Anopheles quadriannulatus SANGWE AQUA001073
Anopheles merus MAF AMEM009095
Anopheles melas CM1001059 A AMEC001158
Anopheles christyi ACHKN1017 ACHR002979
Anopheles epiroticus Epiroticus2 AEPI008195
Anopheles culicifacies A37 ACUA012243
Anopheles minimus MINIMUS1 AMIN006193
Anopheles funestus FUMOZ AFUN016489
Anopheles maculatus maculatus3 AMAM023491
Anopheles dirus WRAIR2 ADIR011242
Anopheles farauti FAR1 AFAF019090
Anopheles atroparvus EBRO AATE008085
Anopheles sinensis SINENSIS ASIS012459
Anopheles albimanus STECLA AALB002022
YTHDF1 Homo sapiens NP_060268.2
YTHDF2 Homo sapiens NP_001166299.1
YTHDF3 Homo sapiens NP_689971.4
CG6422 Drosophila melanogaster AAF56381.1
Anopheles albimanus STECLA AALB003125
Anopheles darlingi Coari ADAC005995
Anopheles atroparvus EBRO AATE019590
Anopheles sinensis China ASIC002272
Anopheles sinensis SINENSIS ASIS020849
Anopheles dirus WRAIR2 ADIR006529
Anopheles farauti FAR1 AFAF005972
Anopheles arabiensis Dongola AARA009973
Anopheles quadriannulatus SANGWE AQUA001395
Anopheles coluzzii Ngousso ACON003817
Anopheles funestus FUMOZ AGAP003817
Anopheles merus MAF AMEM002411
Anopheles melas CM1001059 A AMEC015640
Anopheles christyi ACHKN1017 ACHR000388
Anopheles epiroticus Epiroticus2 AEPI002769
Anopheles culicifacies A37 ACUA004301
Anopheles minimus MINIMUS1 AMIN002883
Anopheles funestus FUMOZ AFUN001584
Anopheles stephensi Indian ASTEI00858
Anopheles stephensi SDA500 ASTE009181
0.0
0.4
0.8
1.2
1.6
2.0
2.4
2.8
3.2
3.6
4.0
4.4
4.8
5.2
5.6
